# Safety and tolerability of elinzanetant: a pooled analysis from four randomized clinical trials

**DOI:** 10.1016/j.eclinm.2026.104250

**Published:** 2026-10-07

**Authors:** JoAnn V. Pinkerton, Maja Francuski, Rossella E. Nappi, James A. Simon, Chenshuang Lu, Senka Djordjevic, Christian Seitz, Nick Panay

**Affiliations:** a Department of Obstetrics and Gynecology, Division Midlife Health, University of Virginia Health, Charlottesville, VA, USA; b Bayer AG, Berlin, Germany; c Department of Clinical, Surgical, Diagnostic and Pediatric Sciences, University of Pavia, Pavia, Italy; d Research Center for Reproductive Medicine, Gynecological Endocrinology and Menopause, IRCCS San Matteo Foundation, Pavia, Italy; e George Washington University, IntimMedicine Specialists, Washington, DC, USA; f Bayer US LLC, Whippany, NJ, USA; g Bayer CC AG, Basel, Switzerland; h Charité - Universitätsmedizin Berlin, Berlin, Germany; i Queen Charlotte's and Chelsea Hospital, Imperial College London, London, UK

**Keywords:** Elinzanetant, Safety, Vasomotor symptoms, Menopause, Hot flashes, Neurokinin

## Abstract

**Background:**

Elinzanetant is a dual neurokinin (NK)-1 and NK-3 receptor antagonist approved for the treatment of moderate-to-severe vasomotor symptoms (VMS) associated with menopause, or those caused by adjuvant endocrine therapy for breast cancer treatment.

**Methods:**

In this pooled safety analysis, safety data were pooled from four randomized, placebo-controlled studies: the phase IIb SWITCH-1 trial (2018–2019) and phase III OASIS-1–3 trials (2021–2024). All four studies were multicentre, multinational, double-blind trials that included postmenopausal women aged 40–65 years experiencing moderate-to-severe VMS due to natural or surgical menopause. Data were analysed exploratively. Assessments included frequency of treatment-emergent adverse events (TEAEs) over 12 and 52 weeks, and exposure-adjusted incidence rates (EAIRs, with 95% confidence intervals [CIs]) over 52 weeks. EAIRs were calculated by weighting event counts against total exposure time (person-years) across studies. Additionally, liver safety and incidence of neoplasms were evaluated.

**Findings:**

The 12-week pooled safety analysis set included 1519 participants: 765 participants in the elinzanetant 120 mg group and 754 participants in the placebo group. The 52-week pooled safety analysis set included 1113 participants in the elinzanetant 120 mg group and 754 participants in the placebo group. TEAEs occurred in 50·8% and 43·2% of participants in the elinzanetant 120 mg and the placebo groups, respectively, over 12 weeks; EAIRs were 196·61 (95% CI 181·33–213·18) and 209·00 (95% CI: 188·96–231·15) in the elinzanetant and placebo groups, respectively, over 52 weeks. The most common TEAEs reported with elinzanetant were headache, fatigue, and somnolence. Treatment discontinuation was low with elinzanetant (7·8%) but higher than placebo (3·6%) over 12 weeks; EAIRs were 14·95 (95% CI 11·99–18·62) and 10·96 (95% CI 7·87–15·27), respectively, over 52 weeks. There were no Hy's Law cases or indication of cholestatic injury. EAIRs of malignant tumors had overlapping CIs between treatment groups. No endometrial hyperplasia/malignancy was reported in either group.

**Interpretation:**

The overall safety profile of elinzanetant appeared favorable in 1113 women based on this exploratory pooled safety analysis. Future time-to-event analyses of TEAEs and real-world post-marketing data are warranted to better characterize the long-term safety profile of elinzanetant.

**Funding:**

Bayer.

**Research in context**

**Evidence before this study**

We searched PubMed from database inception to February 2, 2026, without language restrictions, for randomized clinical trials, meta-analyses, and systematic reviews examining safety outcomes of neurokinin (NK)-targeted therapies (NKTs) for the treatment of vasomotor symptoms (VMS, also called hot flashes/flushes) associated with menopause in postmenopausal women using the following search terms: (“neurokinin” OR “NK-1” OR “NK-3”) AND (“vasomotor symptoms” OR “VMS” OR “hot flash” OR “hot flush”) AND (“safety”) AND (“menopause” or “postmenopause”). This search yielded 26 results; 15 clinical trial papers and 11 meta-analyses/systematic reviews published from 2019 to present. Search results included data on NK-3 receptor antagonist fezolinetant and dual NK-1 and NK-3 receptor antagonist elinzanetant (the focus of this manuscript). A key safety outcome with selective NK-3 antagonist fezolinetant included rare hepatic transaminase ALT/AST elevations in clinical trials and postmarketing cases of hepatotoxicity and jaundice. The postmarketing cases underpin the FDA boxed warning for baseline hepatic screening and routine monitoring of liver function tests in multiple countries. Additionally, a numerical increase in neoplasms was reported in the fezolinetant arm of the SKYLIGHT-4 trial compared to placebo. Although subsequent investigations of malignancy risk with fezolinetant (post hoc, pooled analysis, meta-analysis) found no support for a drug-related increase in neoplasm risk, there is a need for continued ongoing monitoring. No indication for potential serious drug-induced liver injury nor neoplasm risk was identified in Phase III randomized, placebo-controlled trials (OASIS-1, -2, -3, -4) of elinzanetant, and this is reflected in the recently approved labels across multiple global regions.

**Added value of this study**

This study assessed the safety of elinzanetant in a large, pooled dataset (Phase IIb SWITCH-1; Phase III OASIS-1, -2, -3), increasing the likelihood of detecting patterns in safety signals or rare adverse events. OASIS-1, -2, and -3 enrolled postmenopausal women with natural or surgical menopause, whereas OASIS-4 included women receiving endocrine therapy for hormone receptor-positive breast cancer or its prevention in those at high risk for breast cancer. Based on the substantially different study population of patients with breast cancer, data from OASIS-4 study were not included in this pooled safety analysis. Although this post-hoc analysis was exploratory in nature, the safety findings were consistent with those previously characterized in the individual contributing studies, further supporting the overall tolerability of elinzanetant in postmenopausal women. Particularly in areas of special interest, such as liver and endometrial safety, there were no direct indications of hepatotoxicity, and no cases of endometrial malignancy were reported. Within the limitations of no formal direct comparison and the enrolled population with protocol-defined inclusion and exclusion criteria, no increased incidence of neoplasms was observed with use of elinzanetant (up to 52 weeks). Most treatment-emergent adverse events were mild or moderate.

**Implications of all the available evidence**

These safety data are informative for women, prescribers, and payers on the risk–benefit profile of elinzanetant for the treatment of moderate-to-severe VMS associated with natural or surgical menopause. The pooled safety profile of elinzanetant provides the basis for informed decision-making for both HCPs and women with menopause symptoms. Elinzanetant is also approved in Europe for treatment of moderate-to-severe VMS associated with endocrine therapy for breast cancer based on results from OASIS-4, an optional extension of this study is currently ongoing to assess long-term safety of elinzanetant in this population. Future studies incorporating time-to-event analyses of TEAEs and post-marketing real-world data are warranted to better characterize the long-term clinical implications of elinzanetant-associated adverse events.

## Introduction

Neurokinin-targeted therapy (NKT) is a novel drug class for treatment of vasomotor symptoms (VMS) that targets hypothalamic neurokinin receptor activity.^1^^,^^2^ Elinzanetant is the first dual NK-1 and NK-3 receptor antagonist approved for the treatment of moderate-to-severe VMS associated with menopause, and for those caused by adjuvant endocrine therapy related to breast cancer in multiple countries.^3^, ^4^, ^5^, ^6^ Safety outcomes were assessed in the individual clinical trials,^7^, ^8^, ^9^ but an integrated analysis in a large group of participants provides a more comprehensive assessment of the overall safety profile.

VMS, also known as hot flashes (or night sweats, if occurring at night), and sleep disturbances are among the most frequent and disruptive menopause symptoms^1^^,^^10^^,^^11^ and can lead to work and activity impairment, reduced health-related quality of life (QoL), cognitive impairment, and higher healthcare resource utilization and costs.^12^ Elinzanetant has demonstrated rapid, clinically relevant, and sustained efficacy in reducing the frequency and severity of moderate-to-severe VMS associated with natural or surgical menopause versus placebo in the pivotal 26-week Phase III clinical trials, OASIS-1 and -2,^7^ the long-term 52-week Phase III OASIS-3 trial,^8^ and in women taking endocrine therapy for breast cancer in the 52-week OASIS-4 trial.^13^ Elinzanetant also significantly improved sleep disturbance and menopause-related QoL with a favorable safety and tolerability profile.^7^, ^8^, ^9^^,^^13^

Menopause can affect many different organ systems, increase the risk of metabolic and cardiovascular diseases, and affect bone and mental health.^14^ Treatment options for women, therefore, must be evaluated carefully for short-term side effects, contraindications, and longer-term treatment risks.^10^ Menopause hormone therapy (MHT) is an effective treatment for VMS for many women, and the safety profile is generally considered favorable, particularly for women aged <60 years and within 10 years of menopause who have no contraindications.^15^ However, factors such as dosing, type and route of MHT, onset of symptom relief, and individual risk profile are important to consider. The dosing often requires titration to the lowest effective dose based on symptom improvement and tolerability, and maximum relief from symptoms is typically achieved by 8–12 weeks.^16^ MHT can increase risk of endometrial hyperplasia or endometrial cancer in women with intact uterus if the estrogen effect is not adequately antagonized.^10^^,^^12^ The Women's Health Initiative study found that long-term use of only one dosing regimen of an oral MHT was associated with increased risk of breast cancer, stroke, coronary heart disease (CHD), and venous thromboembolic events, when initiated in older women.^15^^,^^17^ Oral and transdermal MHT are contraindicated in women with history of hormone-sensitive cancers (e.g., breast cancer); prior venous thromboembolism, CHD, or stroke; liver disease; and personal history or inherited risk of thromboembolic disease.^15^

Until recently, the only US Food and Drug Administration (FDA)-approved non-hormone treatment option for VMS was the selective serotonin reuptake inhibitor (SSRI) paroxetine.^18^ Most other non-hormone treatment options for VMS currently include drugs approved for other indications that are used off-label.^12^ These have demonstrated modest efficacy in reducing VMS frequency in clinical trials.^1^^,^^10^^,^^19^ Some SSRIs and selective norepinephrine receptor reuptake inhibitors are associated with drowsiness, weight gain, decreased libido, hypertension, constipation, and have poor tolerability with a high discontinuation rate of up to 50% at 3 months.^1^^,^^10^^,^^19^ Longer-term safety of the new drug class of approved NKTs for moderate-to-severe VMS, NK-3 receptor antagonist fezolinetant and the dual NK-1 and NK-3 receptor antagonist elinzanetant, is of interest for practitioners and other stakeholders.^10^^,^^12^^,^^20^

Liver-related health is particularly relevant for women as it can be negatively affected after menopause due to age, hormonal factors, and increasing obesity leading to increased risk of liver diseases and related complications.^21^^,^^22^ There is also emerging evidence of association between severe menopause symptoms and increase in risk of harmful alcohol consumption as a coping mechanism that may further affect liver health.^23^ In addition, prior observation of liver safety concerns with NK-3 receptor antagonists have been observed.^2^ The clinical development of NK-3 receptor antagonist pavinetant was discontinued in 2017 due to transient rise in transaminases in the Phase II trial.^2^ Fezolinetant carries the risk of rare but serious liver injury, leading to an FDA boxed warning for hepatotoxicity and the requirement for multiple follow-up hepatic laboratory testing with its use in multiple countries.^2^^,^^24^^,^^25^ It is, therefore, essential to closely monitor liver enzymes elevations with fezolinetant. The independent liver safety monitoring board (LSMB) concluded that there did not appear to be a significant hepatotoxicity signal with elinzanetant in the Phase III OASIS-1–3 trials,^7^^,^^8^ and no clinically relevant cases of elevated transaminases were reported in Phase IIb SWITCH-1 trial.^9^ Most countries do not require liver monitoring with elinzanetant.^5^^,^^6^^,^^26^ The FDA is the exception; liver monitoring is required prior to and 3 months after initiation of treatment with elinzanetant.^3^

A separate safety concern of potential neoplasm risk in postmenopausal women was investigated for the NK-3 receptor antagonist fezolientant^27^, ^28^, ^29^; however, subsequent investigations including regulatory assessments, post-hoc, and pooled safety data from multiple clinical trials did not confirm any increased cancer risk,^29^, ^30^, ^31^ and no related mechanism was identified.^31^ Additionally, its efficacy in treating VMS is currently being evaluated in women with breast cancer receiving hormone therapy. To date, safety data for elinzanetant relating to endometrial and breast cancer are reassuring.^7^^,^^8^^,^^13^ No cases of endometrial hyperplasia or malignant neoplasm were reported from endometrial biopsies in women treated with elinzanetant in the OASIS-1, -2, and -3 trials or in women with hormone-receptor positive breast cancer in the OASIS-4 trial based on the majority read of three independent blinded external pathologists.^7^^,^^8^^,^^13^ Similarly, no indications of treatment-related safety concerns have been reported from mammograms in the OASIS-1, -2, and -3 trials, and comparable safety profile with placebo has been observed for 52 weeks in a high-risk breast cancer population in the OASIS-4 trial.^7^^,^^8^^,^^13^ Notably, NK-1 receptor antagonists have demonstrated anti-tumor activity in many cancers via inhibition of tumor proliferation, metastasis, and angiogenesis.^32^

This post-hoc and exploratory pooled safety analysis was conducted in participants from Phase IIb SWITCH-1 and Phase III OASIS-1–3 placebo-controlled trials to assess elinzanetant's long-term safety and tolerability in a large group of participants over 52 weeks. Only the studies with similar populations (SWITCH-1, OASIS-1, -2, and -3) were included in the pooled analysis.

## Methods

### Study design, ethics, and participants

In this pooled safety analysis, safety data were pooled from four randomized, placebo-controlled studies: the phase IIb SWITCH-1 trial (2018–2019) and phase III OASIS-1–3 trials (2021–2024). The Phase IIb SWITCH-1 and Phase III OASIS-1, -2, and -3 studies have been described previously (ClinicalTrials.gov identifiers: SWITCH-1: NCT03596762; OASIS-1: NCT05042362, OASIS-2: NCT05099159, OASIS-3: NCT05030584).^7^, ^8^, ^9^ Briefly, all four studies were multicenter, multinational, double-blind, placebo-controlled and included postmenopausal women aged 40–65 years who self-reported their sex as female and were experiencing moderate-to-severe VMS due to natural or surgical menopause. Moderate-to-severe VMS frequency varied across the four studies (Fig. 1).

**Fig. 1:**
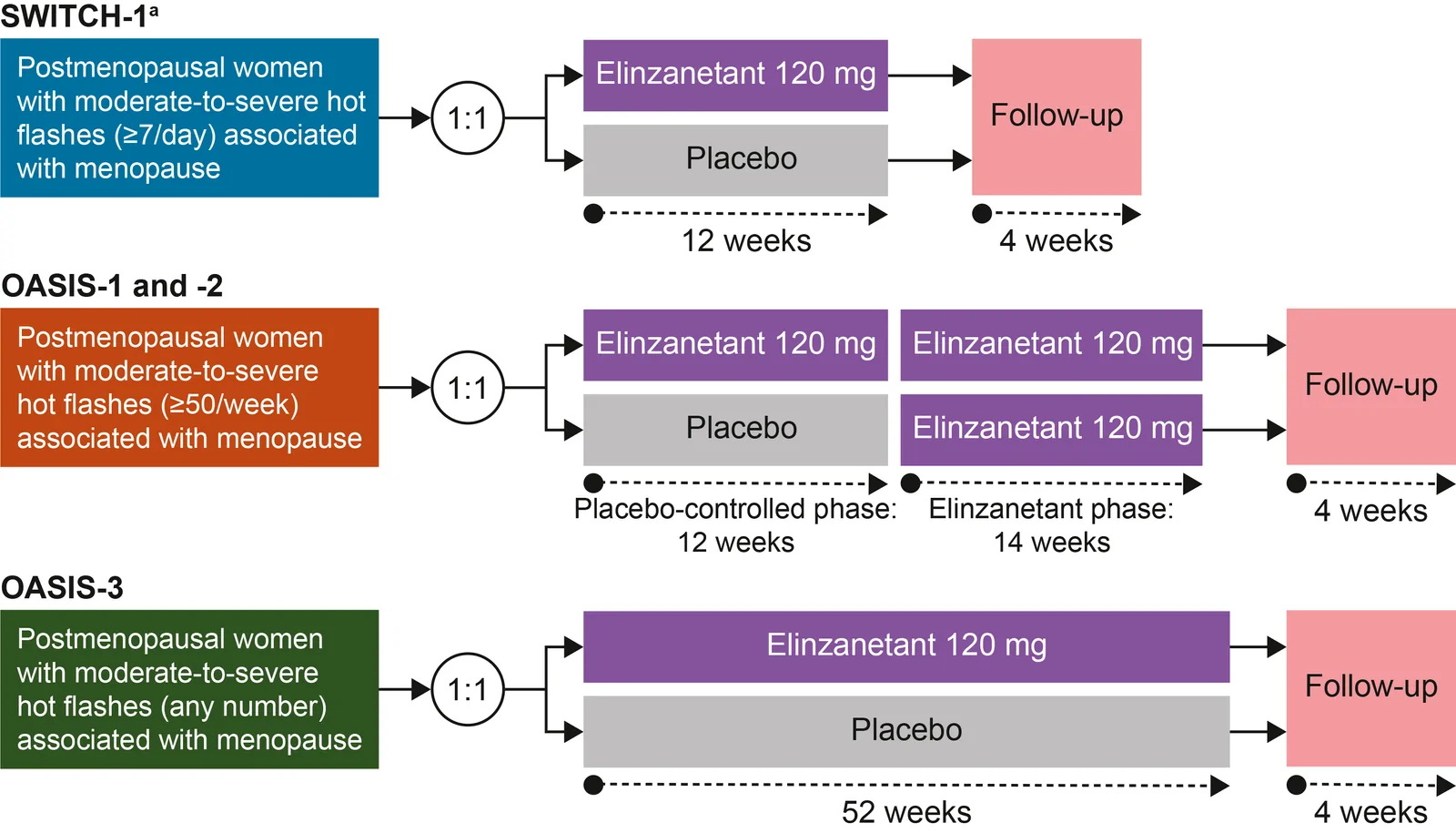
**Study design**. ^a^For SWITCH-1, elinzanetant 40 mg, 80 mg, and 160 mg were excluded from the pooled safety analysis.

This post-hoc pooled safety analysis did not involve any new data collection, contact with participants, or intervention. All four individual trials contributing data to this analysis were conducted in accordance with the Declaration of Helsinki and the Council for International Organizations of Medical Sciences, International Ethical Guidelines and reported using the CONSORT reporting guideline. Institutional review board and ethics committee approvals were obtained for all study sites, and all participants provided written informed consent, as documented in the published studies.^7^, ^8^, ^9^ The written informed consent included secondary use of data for further scientific research and publication of results in scientific articles. No additional consent waiver was sought. Additional details on the study design of four included clinical studies are described in the Supplementary Methods.

Data were pooled from these studies and analyzed over two time periods: placebo-controlled Week 1–12 period and Week 1–52 to include all available data. For the Week 1–12 analysis, data were pooled for the first 12 weeks of treatment, during which all studies had the same treatment duration and included participants treated with elinzanetant versus placebo without any treatment switches. This covered the complete treatment period for SWITCH-1, the first 12 weeks for OASIS-1 and -2, and for OASIS-3, events up to Day 84 after start of first study drug and visit-based measurements up to and including study visit Week 12. The Week 1–52 analysis included participants as treated during the first 12 weeks or after the treatment switch in OASIS-1 and -2 and covered the complete study treatment periods across all studies. For treatment switchers, events and measurements were only assigned to one treatment, namely the one that was given when the event started, or the measurement was collected.

### TEAEs

Adverse events (AEs) were reported by the participants. The investigators and other qualified healthcare professionals were responsible for detecting, documenting, and recording AEs, which were coded using the Medical Dictionary for Regulatory Activities (MedDRA) version 26·1. A treatment-emergent adverse event (TEAE) was defined as any AE occurring or worsening on or after the date of the first dose and up to 14 days after the last dose. AE seriousness, intensity, and relatedness to the study intervention were assessed by the investigators. For the first 12 weeks, TEAEs that started in the placebo or elinzanetant phase were assigned to the respective group. For the 52 weeks analysis, the elinzanetant group included all TEAEs that occurred during the elinzanetant exposure period among all participants who received elinzanetant, i.e., including treatment switchers if they switched from placebo to elinzanetant, whereas the placebo group only included TEAEs that occurred during the 12-week placebo phase. AEs of special interest included any condition triggering close liver observation or increase in liver enzymes meeting the following criteria: alanine transaminase (ALT) and/or aspartate transaminase (AST) > 8 times the upper limit of normal (ULN), or ALT and/or AST >3 times ULN and total bilirubin (TBL) > 2 times ULN; somnolence or fatigue; photosensitivity; and postmenopausal bleeding.

### Liver safety assessments

Hepatic safety monitoring for the Phase IIb SWITCH-1 and Phase III OASIS-1, OASIS-2, and OASIS-3 trials has been described previously.^33^ Closer liver observation criteria and independent blinded review by external LSMB for cases meeting the predefined close liver observation criteria to identify any potential drug-induced liver injury (DILI) were also described previously.^33^ The criteria were AST or ALT levels at more than 3 × ULN or alkaline phosphatase (ALP) levels at more than 2 × ULN (as confirmed by retest). In SWITCH-1, only increases in liver enzymes ≥3 × ULN were considered relevant per FDA guidance.^9^ Close liver observation cases were not defined in SWITCH-1 and were excluded from this analysis. It was reported that there was no evidence of drug-related liver toxicity in SWITCH-1, with sporadic increase in transaminases showing equal frequency across elinzanetant and placebo groups and considered not clinically relevant.^9^

### Neoplasms and malignant tumors

Additional post-hoc analyses were conducted to identify TEAEs and post-treatment adverse events (PTAEs) using primary system organ class (SOC): neoplasms benign, malignant and unspecified (including cysts and polyps), and Standardized MedDRA queries (SMQ): malignant tumors or premalignant disorders, general conditions and other site-specific disorders, and breast malignant tumors. Post-treatment refers to the time after the last study drug was given and are only available for the Week 1–12 treatment groups. PTAEs in participants switching from placebo to elinzanetant, which occurred after the 14-day TEAE window after the last elinzanetant treatment, were included in placebo group (Week 1–12). The exposure-adjusted incidence rates (EAIRs) for malignant tumors and breast cancer were compared to the background incidence rates obtained from real-world data in US women aged 50–64 years from Surveillance, Epidemiology, and End Results (SEER) program.^34^

Findings from endometrial biopsies reported in individual clinical studies were not pooled for this analysis because of different treatment duration. The assessment of endometrial safety for FDA is typically performed on 1-year biopsies.^35^ This treatment duration was only achieved in OASIS-3.

Other safety assessments are described in the Supplementary Methods.

### Statistical analysis

The analyses presented are exploratory in nature. They are based on an integrated summary of safety data and are not powered or designed for formal hypothesis testing, which could potentially mislead the interpretation of results. Therefore, no sample size was calculated. In addition, treatment durations differed across studies, which may have affected the opportunity to observe events and could bias direct between-group comparisons. The results were summarized descriptively using exposure-adjusted incidence rates (EAIRs), with 95% confidence interval (CI). The methodology by which EAIRs and CIs were calculated is described in the Supplementary Methods. Mantel-Haenszel risk difference between elinzanetant 120 mg (Week 1–12) and placebo (Week 1–12) was stratified by study. No formal comparison was made between treatment groups for risk difference analysis, and the reported values are exploratory and non-confirmatory in nature.

### Role of the funding source

Bayer had a role in the design and conduct of the pooled analysis; collection, management, analysis, and interpretation of the data; preparation, review, or approval of the manuscript; and decision to submit the manuscript for publication.

## Results

### Demographics

Participant disposition in the overall study is summarized in Fig. 2. The 12-week pooled safety analysis set included 1519 participants: 765 participants in the elinzanetant 120 mg group and 754 participants in the placebo group. The 52-week pooled safety analysis set included 1113 participants in the elinzanetant 120 mg group and 754 participants in the placebo group.

**Fig. 2:**
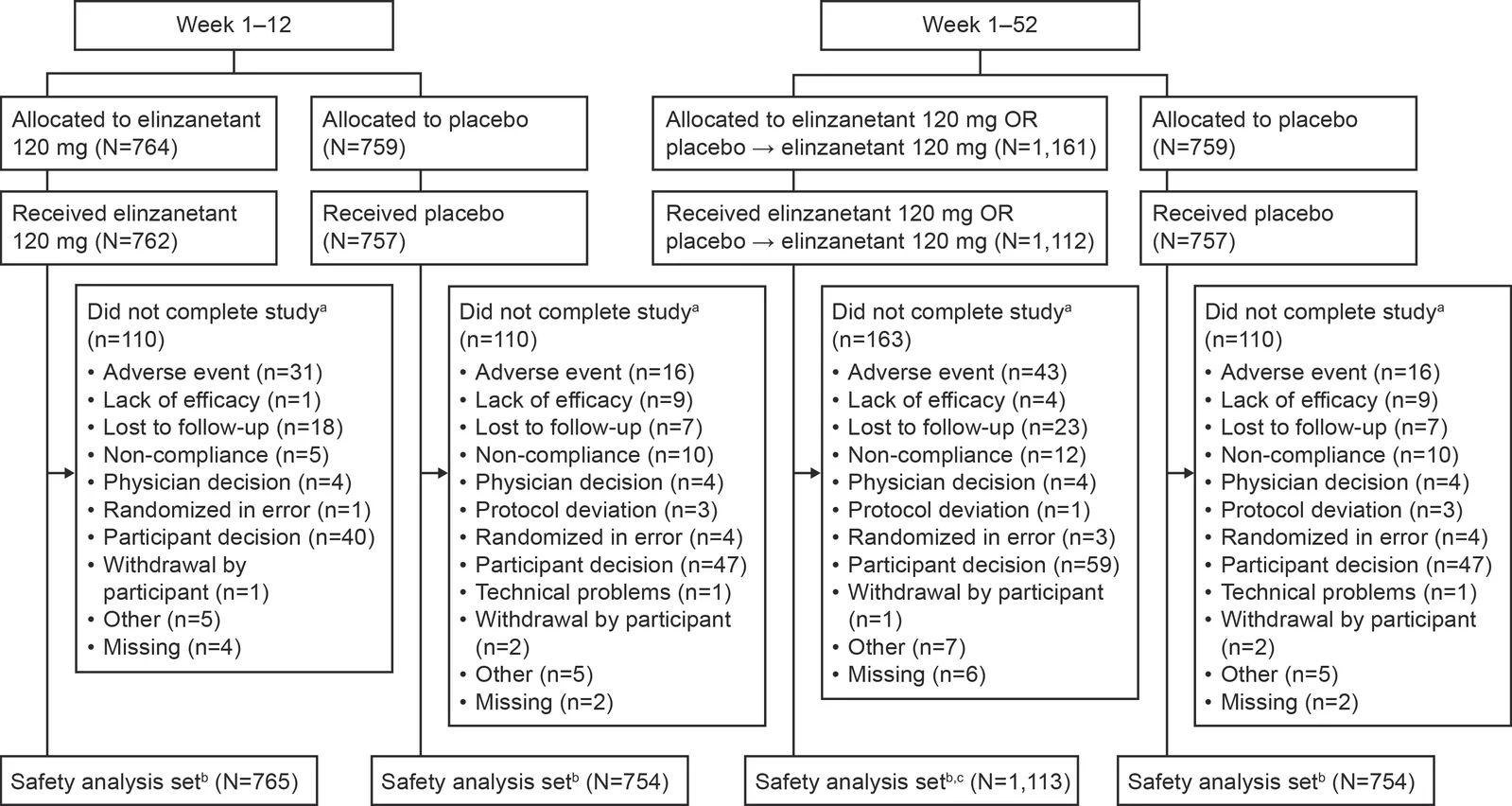
**Participants disposition in overall study (all randomized participants)**. ^a^Does not include participants who discontinued the study drug but remained in the study and completed the post-treatment phase/follow-up. ^b^In the safety analysis set, participants were analyzed according to the actual treatment they received, irrespective of the randomized allocation. ^c^Participants exposed to elinzanetant 120 mg or those who switched to elinzanetant 120 mg at some point during the OASIS-1 and -2 studies.

Demographic and baseline characteristics were generally well balanced between the elinzanetant and placebo groups in the Week 1–12 analysis and with all the participants treated with elinzanetant 120 mg in the Week 1–52 analysis. Overall, the mean (SD) age varied from 54·6 to 54·7 (4·8) years and most of the participants were in the 50–59 age group. Mean (SD) body mass index (BMI) varied from 27·63 (4·71) to 27·78 (4·65) and the median (min, max) BMI between 27·20 and 27·25 (40, 65). Participants were evenly distributed across different BMI groups except for a few participants with BMI <18·5. Participants were predominantly White (77·5%–81·6%) and Black or African American (14·7%–17·8%). Few were Hispanic or Latino (8·8%–9·7%), and majority of the participants had never smoked. Similar proportion of participants had undergone hysterectomy (33·1%–34·8%) and oophorectomy (15·3%–18·6%), and the mean (SD) duration of amenorrhea was similar across all the treatments groups (Table 1).

**Table 1:** Baseline demographic characteristics—SWITCH-1 and OASIS-1–3.

Characteristic	Week 1–12		Week 1–52	
	Elinzanetant 120 mg N = 765	Placebo N = 754	Elinzanetant 120 mg^a^ N = 1113	Placebo N = 754
Age, mean (SD), years	54·7 (4·8)	54·7 (4·8)	54·6 (4·8)	54·7 (4·8)
Age group, n (%)				
40–49 years	104 (13·6)	104 (13·8)	156 (14·0)	104 (13·8)
50–59 years	520 (68·0)	519 (68·8)	761 (68·4)	519 (68·8)
60–65 years	141 (18·4)	131 (17·4)	196 (17·6)	131 (17·4)
BMI (kg/m^2^), mean (SD)	27·63 (4·71)	27·78 (4·65)	27·67 (4·67)	27·78 (4·65)
BMI (kg/m^2^) group, n (%)				
<18·5	5 (0·7)	4 (0·5)	6 (0·5)	4 (0·5)
18·5–<25	245 (32·0)	223 (29·6)	350 (31·4)	223 (29·6)
25–<30	279 (36·5)	291 (38·6)	405 (36·4)	291 (38·6)
≥30	236 (30·8)	236 (31·3)	352 (31·6)	236 (31·3)
Race, n (%)				
White	593 (77·5)	615 (81·6)	883 (79·3)	615 (81·6)
Black or African American	136 (17·8)	111 (14·7)	187 (16·8)	111 (14·7)
Asian	5 (0·7)	5 (0·7)	6 (0·5)	5 (0·7)
American Indian or Alaska Native	2 (0·3)	2 (0·3)	4 (0·4)	2 (0·3)
Native Hawaiian or Other Pacific Islander	1 (0·1)	0	1 (<0·1)	0
Multiple	4 (0·5)	2 (0·3)	5 (0·4)	2 (0·3)
Other	0	1 (0·1)	0	1 (0·1)
Not reported	24 (3·1)	18 (2·4)	27 (2·4)	18 (2·4)
Ethnicity, n (%)				
Not Hispanic or Latino	682 (89·2)	668 (88·6)	991 (89·0)	668 (88·6)
Hispanic or Latino	67 (8·8)	73 (9·7)	102 (9·2)	73 (9·7)
Not reported	16 (2·1)	13 (1·7)	20 (1·8)	13 (1·7)
Smoking history, n (%)				
Never	445 (58·2)	435 (57·7)	661 (59·4)	435 (57·7)
Former	139 (18·2)	138 (18·3)	203 (18·2)	138 (18·3)
Current	129 (16·9)	134 (17·8)	197 (17·7)	134 (17·8)
Duration of amenorrhea, mean (SD), years	7·04 (6·25)	7·03 (6·44)	6·97 (6·20)	7·03 (6·44)
Hysterectomy,^b^ n (%)	253 (33·1)	259 (34·4)	387 (34·8)	259 (34·4)
Oophorectomy,^c^ n (%)	117 (15·3)	140 (18·6)	193 (17·3)	140 (18·6)

BMI, body mass index; SD, standard deviation.

a Participants exposed to elinzanetant 120 mg or those who switched to elinzanetant 120 mg at some point during the OASIS-1 and -2 studies.

b Included all participants who had medical history of hysterectomy, hysterosalpingectomy, hysterosalpingo-oophorectomy, or radical hysterectomy.

c Included all participants who had a medical history of oophorectomy, bilateral oophorectomy, hysterosalpingo-oophorectomy, salpingo-oophorectomy, bilateral salpingo-oophorectomy, or unilateral salpingo-oophorectomy.

### Summary of TEAEs

The proportion of participants with overall TEAEs was 50·8% in the elinzanetant 120 mg group and 43·2% in the placebo group in the first 12 weeks. The EAIRs were 196·61 (95% CI 181·33–213·18) and 209·00 (95% CI 188·96–231·15) in the elinzanetant 120 mg and placebo groups, respectively, over 52 weeks. In most participants, TEAEs were reported as mild or moderate in intensity. Treatment-related TEAEs were reported in a higher proportion of participants in the elinzanetant 120 mg group compared with placebo group both during the 12- and 52-week periods (Week 1–12 elinzanetant 120 mg: 22·6%, placebo: 10·7%; Week 1–52 elinzanetant 120 mg: 20·5% [EAIR: 48·95, 95% CI 42·82–55·95], placebo: 13·0% [EAIR: 35·92, 95% CI 29·60–43·58]). Overall, serious TEAEs were reported in a small number of participants in both groups. Serious TEAEs occurred in 1·2% and 0·9% of participants in the elinzanetant 120 mg and placebo groups during the first 12 weeks, with EAIRs of 5·46 (95% CI 3·80–7·85) and 2·92 (95% CI 1·55–5·51) during the 52-week period, respectively (Table 2). One serious TEAE (generalized tonic-clonic seizure) that occurred in one (<0·1%) participant among three (0·3%) participants with epilepsy in the elinzanetant 120 mg group (Week 1–52) was assessed by the investigator as related to study intervention. This participant had a long history of generalized tonic-clonic seizures and discontinued the study. All remaining serious TEAEs were assessed by the investigator as not related to treatment (Supplementary Table S1). The overall incidence of TEAEs leading to discontinuation was low; the proportion of participants discontinuing due to TEAEs was higher with elinzanetant 120 mg compared with placebo (Table 2). Specifically, TEAEs leading to discontinuation occurred in 7·8% and 7·5% (EAIR: 14·95, 95% CI 11·99–18·62) participants over 12 and 52 weeks in the elinzanetant 120 mg group versus 3·6% and 4·1% (EAIR: 10·96, 95% CI 7·87–15·27) in the placebo group. The most frequent contributing categories up to 12 weeks included: gastrointestinal disorders (2·1% versus 1·5%); nervous system disorders (2·4% versus 1·1%); and general disorders and administration site conditions (1·8% versus 0%) in the elinzanetant 120 mg and placebo groups, respectively. The most frequent contributing categories up to 52 weeks included: gastrointestinal disorders (2·1% versus 1·7%); nervous system disorders (2·0% versus 1·2%); and general disorders and administration site conditions (1·3% versus 0%) in the elinzanetant 120 mg and placebo groups, respectively. The frequency of participants reporting treatment discontinuation with elinzanetant did not increase over 52 weeks, suggesting that most discontinuations occurred during the first 12 weeks. Photosensitivity was reported by a small number of participants in the elinzanetant 120 mg group (Week 1–12: 2 [0·3%], Week 1–52: 5 [0·4%, EAIR 0·86, 95% CI 0·36–2·09]) versus no participant in placebo in Week 1–12 and one (0·1%, EAIR 0·22, 95% CI 0·03–1·61) participant in Week 1–52. The cases of photosensitivity were reported either as photosensitivity, or itchy skin on sun-exposed skin areas, redness/sunburn, or phototoxicity. Time to onset varied between 1 and 290 days. Postmenopausal uterine bleeding occurred at very low rates and were generally comparable in the elinzanetant 120 mg and the placebo group over 12 weeks (elinzanetant: 2·1% versus placebo: 2·0%) and 52 weeks (elinzanetant: 2·1%, EAIR 4·17, 95% CI 2·52–6·91 versus placebo: 3·0%, EAIR 6·35, 95% CI 3·76–10·71).

**Table 2:** Overview of TEAEs—SWITCH-1 and OASIS-1–3.

	Week 1–12			Week 1–52			
	Elinzanetant 120 mg (N = 765)	Placebo (N = 754)	Risk difference (95% CI)^a^	Elinzanetant 120 mg^b^ (N = 1113)		Placebo (N = 754)	
	n (%)			n (%)	EAIR^c^ (95% CI)^d^	n (%)	EAIR^c^ (95% CI)^d^
Any TEAE	389 (50·8)	326 (43·2)	7·55 (2·56–12·54)	614 (55·2)	196·61 (181·33–213·18)	387 (51·3)	209·00 (188·96–231·15)
Any study drug–related TEAE	173 (22·6)	81 (10·7)	11·89 (8·20–15·58)	228 (20·5)	48·95 (42·82–55·95)	98 (13·0)	35·92 (29·60–43·58)
Any TEAE leading to discontinuation of study drug	60 (7·8)	27 (3·6)	4·29 (1·97–6·60)	83 (7·5)	14·95 (11·99–18·62)	31 (4·1)	10·96 (7·87–15·27)
Any serious TEAE	9 (1·2)	7 (0·9)	0·25 (−0·78 to 1·27)	31 (2·8)	5·46 (3·80–7·85)	10 (1·3)	2·92 (1·55–5·51)
Any study drug–related serious TEAE	0	0	–	1 (<0·1)	0·14 (0·02–0·98)	0	–

CI confidence interval; EAIR, exposure-adjusted incidence rate; TEAE, treatment-emergent adverse event.

N = number of subjects who received the respective treatment at any time, n = number of subjects with at least one such event.

a Mantel-Haenszel risk difference between elinzanetant 120 mg and placebo stratified by study. If the number of participants with an event was zero in both treatment groups in one study, that study was excluded from the calculation of risk difference. Risk differences are only shown where there were ≥5 participants with one such event in either treatment group.

b Participants exposed to elinzanetant 120 mg or those who switched to elinzanetant 120 mg at some point during the OASIS-1 and -2 studies.

c EAIRs are defined as study size and exposure-adjusted incidence rates according to Crowe et al. (2016).^34^ EAIRs are provided per 100 person-years, where one person-year is defined as 365·25 days.

d The CI for EAIRs was calculated by stratified Wilson CI according to Yan et al. (2010).^35^

### Most common TEAEs

Headache (7·5%), fatigue (5·4%), and somnolence (3·4%) were the most common TEAEs in the elinzanetant 120 mg group during the first 12 weeks and occurred at a higher frequency compared with placebo (headache: 4·2%, fatigue: 1·3%, and somnolence: 0·5%). Headache and fatigue were also the most common TEAEs in the elinzanetant 120 mg group over 52 weeks with EAIRs of 15·48 (95% CI 12·41–19·31) and 9·40 (95% CI 7·10–12·45), respectively, compared with EAIRs of 11·17 (95% CI 8·00–15·59) and 4·73 (95% CI 2·86–7·82) in the placebo group (Table 3). One case of headache in each group was assessed as severe; all other cases of headache, fatigue, and somnolence were mild or moderate. The frequency of participants experiencing headache, fatigue, and somnolence was the same or decreased over 52 weeks compared with 12 weeks in the elinzanetant 120 mg group, suggesting that most of these TEAEs were reported in the first 12 weeks.

**Table 3:** TEAEs by preferred term (≥2% in any treatment group over 52 weeks)—SWITCH-1 and OASIS-1–3.

TEAEs	Week 1–12		Week 1–52			
	Elinzanetant 120 mg (N = 765)	Placebo (N = 754)	Elinzanetant 120 mg^a^ (N = 1113)		Placebo (N = 754)	
	n (%)		n (%)	EAIR^b^ (95% CI)^c^	n (%)	EAIR^b^ (95% CI)^c^
Headache	57 (7·5)	32 (4·2)	84 (7·5)	15·48 (12·41–19·31)	38 (5·0)	11·17 (8·00–15·59)
Fatigue	41 (5·4)	10 (1·3)	52 (4·7)	9·40 (7·10–12·45)	15 (2·0)	4·73 (2·86–7·82)
Somnolence	26 (3·4)	4 (0·5)	27 (2·4)	5·35 (3·70–7·75)	7 (0·9)	2·03 (0·95–4·32)
Arthralgia	23 (3·0)	20 (2·7)	35 (3·1)	6·14 (4·35–8·66)	25 (3·3)	8·51 (5·83–12·41)
Dizziness	23 (3·0)	8 (1·1)	32 (2·9)	5·75 (4·04–8·21)	9 (1·2)	2·93 (1·55–5·52)
COVID-19	21 (2·7)	25 (3·3)	48 (4·3)	8·87 (6·64–11·84)	44 (5·8)	12·92 (9·45–17·65)
Depression rating scale score increased	21 (2·7)	28 (3·7)	25 (2·2)	3·58 (2·29–5·60)	28 (3·7)	13·00 (9·58–17·62)
Nausea	19 (2·5)	14 (1·9)	29 (2·6)	4·96 (3·38–7·26)	19 (2·5)	5·89 (3·75–9·25)
Diarrhea	16 (2·1)	14 (1·9)	34 (3·1)	6·04 (4·27–8·55)	15 (2·0)	5·56 (3·49–8·84)
Gastroesophageal reflux disease	15 (2·0)	5 (0·7)	23 (2·1)	3·67 (2·36–5·71)	9 (1·2)	2·45 (1·23–4·88)
Nasopharyngitis	14 (1·8)	12 (1·6)	40 (3·6)	7·16 (5·20–9·84)	31 (4·1)	8·64 (5·93–12·58)
Urinary tract infection	11 (1·4)	12 (1·6)	29 (2·6)	5·00 (3·42–7·31)	18 (2·4)	4·71 (2·84–7·81)
Back pain	11 (1·4)	6 (0·8)	22 (2·0)	3·99 (2·61–6·10)	13 (1·7)	3·58 (2·01–6·38)
Hypertension	7 (0·9)	14 (1·9)	19 (1·7)	3·61 (2·31–5·63)	15 (2·0)	4·57 (2·73–7·64)

CI, confidence interval; EAIR, exposure-adjusted incidence rate; TEAE, treatment-emergent adverse event.

N = number of subjects who received the respective treatment at any time, n = number of subjects with at least one such event.

a Participants exposed to elinzanetant 120 mg or those who switched to elinzanetant 120 mg at some point during the OASIS-1 and -2 studies.

b EAIRs are defined as study size and exposure-adjusted incidence rates according to Crowe et al. (2016).^34^ EAIRs are provided per 100 person-years, where one person-year is defined as 365·25 days.

c The CI for EAIRs was calculated by stratified Wilson CI according to Yan et al. (2010).^35^

The most frequently reported TEAEs assessed by the investigator as related to elinzanetant were fatigue, headache, and somnolence during both 12- and 52-week periods (Table 4).

**Table 4:** TEAEs by preferred term as assessed by the investigator as study drug–related (≥2% in any treatment group over 52 weeks)—SWITCH-1 and OASIS-1–3.

Study drug–related TEAEs	Week 1–12		Week 1–52			
	Elinzanetant 120 mg (N = 765)	Placebo (N = 754)	Elinzanetant 120 mg^a^ (N = 1113)		Placebo (N = 754)	
	n (%)		n (%)	EAIR^b^ (95% CI)^c^	n (%)	EAIR^b^ (95% CI)^c^
Fatigue	36 (4·7)	5 (0·7)	39 (3·5)	6·78 (4·88–9·42)	7 (0·9)	2·01 (0·94–4·30)
Headache	30 (3·9)	12 (1·6)	37 (3·3)	6·21 (4·41–8·75)	12 (1·6)	4·10 (2·39–7·02)
Somnolence	23 (3·0)	3 (0·4)	24 (2·2)	4·76 (3·22–7·04)	5 (0·7)	1·57 (0·67–3·69)
Dizziness	15 (2·0)	6 (0·8)	19 (1·7)	3·27 (2·05–5·22)	7 (0·9)	2·49 (1·26–4·94)

N = number of subjects who received the respective treatment at any time, n = number of subjects with at least one such event.

CI, confidence interval; EAIR, exposure-adjusted incidence rate; TEAE, treatment-emergent adverse event.

a Participants exposed to elinzanetant 120 mg or those who switched to elinzanetant 120 mg at some point during the OASIS-1 and -2 studies.

b EAIRs are defined as study size and exposure-adjusted incidence rates according to Crowe et al. (2016).^34^ EAIRs are provided per 100 person-years, where one person-year is defined as 365·25 days.

c The CI for EAIRs was calculated by stratified Wilson CI according to Yan et al. (2010).^35^

Fatigue and headache were the most common TEAEs leading to treatment discontinuation in 13 (1·7%) elinzanetant-treated versus zero placebo-treated participants, and 11 (1·4%) elinzanetant-treated versus five (0·7%) placebo-treated participants, respectively, during the first 12 weeks. Somnolence led to treatment discontinuation in two (0·3%) elinzanetant-treated versus zero placebo-treated participants over 12 weeks. There was no additional discontinuation due to fatigue or somnolence beyond 12 weeks in both groups, resulting in an EAIR of 2·23 (95% CI 1·27–3·91) for fatigue and 0·36 (95% CI 0·10–1·34) for somnolence over 52 weeks for elinzanetant 120 mg group; one additional discontinuation was reported for headache in elinzanetant 120 mg group, resulting in an EAIR of 2·01 (95% CI 1·12–3·63), generally comparable to the EAIR of 2·08 (95% CI 0·99–4·37) for placebo group over 52 weeks.

### Liver safety assessments

Hepatic laboratory assessments are summarized in Table 5. Elevated post-baseline values of ALT/AST ≥3 × ULN were observed in four (0·5%) participants in the elinzanetant 120 mg group and three (0·4%) participants in the placebo group over 12 weeks, with EAIR of 2·05 (95% CI 0·80–5·29) and 1·65 (95% CI 0·56–4·85), respectively. There were no ALT or AST elevations ≥5 × ULN or TBL elevations ≥2 × ULN over 12 weeks in either treatment group. Over 52 weeks, elevated post-baseline values of ALT/AST ≥3 × ULN were observed in 13 (1·2%) and seven (1·0%) participants in the elinzanetant 120 mg and placebo groups, respectively, with EAIR of 2·11 (95% CI 1·23–3·63) and 1·62 (95% CI 0·72–3·65). There was a low and generally balanced incidence of AST or ALT elevations ≥5 × ULN across the treatment groups. One participant in the elinzanetant group had a TBL ≥2 × ULN but without ALT or AST elevations ≥3 × ULN at Week 24/26; all other measurements before and after showed normal TBL levels in serum or plasma.

**Table 5:** Hepatic laboratory assessments at any time post-baseline—SWITCH-1 and OASIS-1–3.

Parameter	Week 1–12				Week 1–52			
	Elinzanetant 120 mg (N = 765)		Placebo (N = 754)		Elinzanetant 120 mg^a^ (N = 1113)		Placebo (N = 754)	
	n (%)	EAIR^b^ (95% CI)^c^	n (%)	EAIR^b^ (95% CI)^c^	n (%)	EAIR^b^ (95% CI)^c^	n (%)	EAIR^b^ (95% CI)^c^
ALT								
n	753		735		1098		735	
≥3 × ULN	3 (0·4)	1·54 (0·52–4·55)	2 (0·3)	1·10 (0·30–4·00)	13 (1·2)	2·11 (1·23–3·64)	6 (0·8)	1·42 (0·60–3·38)
≥5 × ULN	0	–	0	–	2 (0·2)	0·32 (0·09–1·19)	2 (0·3)	0·39 (0·08–1·83)
≥8 × ULN^d^	0	–	0		1 (<0·1)	0·19 (0·04–0·98)	1 (0·1)	0·20 (0·03–1·49)
AST								
n	755		735		1100		735	
≥3 × ULN	3 (0·4)	1·54 (0·52–4·54)	1 (0·1)	0·55 (0·10–3·11)	5 (0·5)	0·83 (0·35–1·94)	4 (0·5)	0·78 (0·25–2·44)
≥5 × ULN	0	–	0	–	0	–	1 (0·1)	0·19 (0·03–1·49)
≥8 × ULN^d^	0	–	0	–	0	–	1 (0·1)	0·19 (0·03–1·49)
ALT or AST								
n	755		735		1100		735	
≥3 × ULN	4 (0·5)	2·05 (0·80–5·29)	3 (0·4)	1·65 (0·56–4·85)	13 (1·2)	2·11 (1·23–3·63)	7 (1·0)	1·62 (0·72–3·65)
≥5 × ULN	0	–	0	–	2 (0·2)	0·32 (0·09–1·18)	2 (0·3)	0·39 (0·08–1·83)
≥8 × ULN^d^	0	–	0	–	1 (<0·1)	0·19 (0·04–0·98)	1 (0·1)	0·19 (0·03–1·49)
ALP								
n	755		735		1100		735	
≥1·5 × ULN	6 (0·8)	3·10 (1·42–6·77)	11 (1·5)	6·09 (3·41–10·89)	22 (2·0)	3·38 (2·20–5·21)	16 (2·2)	5·20 (3·27–8·27)
≥2 × ULN	1 (0·1)	0·50 (0·09–2·89)	2 (0·3)	1·12 (0·31–4·03)	4 (0·4)	0·69 (0·28–1·75)	3 (0·4)	0·60 (0·17–2·16)
≥3 × ULN	1 (0·1)	0·50 (0·09–2·89)	0	–	2 (0·2)	0·38 (0·11–1·28)	1 (0·1)	0·19 (0·03–1·49)
Total bilirubin								
n	753		735		1098		735	
≥1 × ULN	24 (3·2)	12·58 (8·45–18·72)	17 (2·3)	9·43 (5·89–15·09)	40 (3·6)	6·01 (4·33–8·34)	19 (2·6)	6·03 (3·92–9·29)
≥2 × ULN^e^	0	–	0	–	1 (<0·1)	0·19 (0·04–0·98)	0	–
INR								
n	703		688		1048		688	
≥1·5	5 (0·7)	2·83 (1·21–6·61)	8 (1·2)	4·80 (2·44–9·45)	18 (1·7)	2·76 (1·70–4·47)	13 (1·9)	3·41 (1·91–6·10)
≥2	2 (0·3)	1·13 (0·31–4·11)	3 (0·4)	1·80 (0·62–5·26)	7 (0·7)	0·97 (0·44–2·16)	5 (0·7)	1·27 (0·50–3·22)

n = number of participants with parameter assessment done at respective time point. Unscheduled visits were included in the analysis.

ALP, alkaline phosphatase; ALT, alanine transferase; AST, aspartate aminotransferase; CI, confidence interval; EAIR, exposure-adjusted incidence rate; ULN, upper limit of normal.

a Participants exposed to elinzanetant 120 mg or those who switched to elinzanetant 120 mg at some point during the OASIS-1 and -2 studies.

b EAIRs are defined as study size and exposure-adjusted incidence rates according to Crowe et al. (2016).^34^ EAIRs are provided per 100 person-years, where one person-year is defined as 365·25 days.

c The CI for EAIRs was calculated by stratified Wilson CI according to Yan et al. (2010).^35^

d No cases of ≥10 × ULN were reported.

e No cases of ≥5 × ULN were reported.

No cases of liver enzyme elevations meeting criteria for liver injury were reported during the first 12 weeks. Over 52 weeks, eight cases in the elinzanetant 120 mg group and four cases in the placebo group fulfilled the criteria for close liver observation. A summary of all the individual cases fulfilling the close liver observation criteria during elinzanetant treatment in the Phase III OASIS-1–3 studies has been published previously.^33^ One case in the elinzanetant 120 mg group and two cases in the placebo group met the liver injury criteria as assessed by LSMB. The causality by study treatment was assessed as possible for one case (alternative etiology: possible passage of gallstones) in the elinzanetant 120 mg group and probable for one case in the placebo group by the LSMB (no alternative etiology). Both cases recovered or resolved, without treatment discontinuation in the elinzanetant group and with treatment interruption in the placebo group. The remaining 10 cases were assessed as unlikely related to treatment in both groups, and most cases recovered or resolved with or without treatment discontinuation. There were no cases of potential Hy's law reported at any time point and one (<0·1%) case of cholestasis reported in the elinzanetant group during the 52-week period with EAIR of 0·19. The cholestasis was reported in the same participant with TBL ≥2 × ULN, but without ALT/AST ≥3 × ULN and ALP below levels of ≥1× ULN. The condition resolved without treatment discontinuation.

### Assessment of neoplasms and malignant tumors

Participants with TEAEs and PTAEs identified in SOC neoplasms benign, malignant, and unspecified are reported in Supplementary Table S2, and those in SMQ malignant tumors and breast malignant tumors are reported in Table 6. No TEAEs or PTAEs in SMQ premalignant disorders, general conditions and other site-specific disorders were collected. Overall, benign and malignant neoplasms were reported in 12 (1·1%, EAIR 1·89 [95% CI 1·07–3·35]) participants in the elinzanetant 120 mg group versus four (0·5%, EAIR 1·02 [95% CI 0·37–2·79]) participants in the placebo group with overlapping CIs. Specifically, for malignant tumors, six and three malignancies were reported in five (0·4%) and two (0·3%) participants in elinzanetant and placebo groups, respectively. While formal direct comparison is limited due to the exploratory nature of the analysis, the corresponding EAIR of 0·88 (95% CI 0·39–2·01) for malignant tumors in the elinzanetant group appeared broadly consistent with population-level incidence rate (IR) of 0·81 (95% CI 0·81–0·82) for all cancer in real-world data from US women aged 50–64 years from the SEER program.^34^ No cases of endometrial hyperplasia or malignancy were reported in either elinzanetant or placebo group over 52 weeks. There were three AEs of breast malignancy reported in two participants in the elinzanetant group, all reported in the OASIS-3 study. The corresponding EAIR of 0·38 (95% CI, 0·12–1·28) was broadly consistent with population-level IR of 0·29 (95% CI 0·28–0·29) for breast cancer in US women aged 50–64 years from SEER program.^34^ No breast malignancy was reported in the placebo group. The reported breast malignancies included invasive ductal breast carcinoma in one participant reported as a serious event, triple negative, and was not considered related to treatment by the investigator. The other participant reported fibroadenoma with suspected malignancy of the right mammary gland that resolved and a low-differentiated, triple-negative invasive breast carcinoma that was reported as a moderate event and the outcome is unknown.

**Table 6:** Participants with TEAEs and PTAEs in SMQ malignant tumors or premalignant disorders, general conditions and other site-specific disorders by preferred term and SMQ breast malignant tumors Week 1–52—SWITCH-1 and OASIS-1–3.

TEAEs and PTAEs	Week 1–52			
	Elinzanetant 120 mg^a^ (N = 1113)		Placebo (N = 754)	
	n (%)	EAIR^b^ (95% CI)^c^	n (%)	EAIR^b^ (95% CI)^c^
Participants with ≥1 event of malignant tumors	5 (0·4)	0·88 (0·39–2·01)	2 (0·3)	0·39 (0·08–1·82)
Preferred term				
Adenocarcinoma of colon	1 (<0·1)	0·12 (0·02–0·86)	0	–
Basal cell carcinoma^d^	0	–	2 (0·3)	0·39 (0·08–1·82)
Bowen's disease	1 (<0·1)	0·18 (0·03–0·96)	0	
Squamous cell carcinoma of skin	1 (<0·1)	0·19 (0·04–0·98)	1 (0·1)	0·19 (0·03–1·49)
SMQ breast malignant tumors^e^	2 (0·2)	0·38 (0·12–1·28)	0	–

N = number of subjects who received the respective treatment at any time, n = number of subjects with at least one such event.

CI, confidence interval; EAIR, exposure-adjusted incidence rate; PTAE, post-treatment adverse event; SMQ, Standardized MedDRA Query; TEAE, treatment-emergent adverse event.

a Participants exposed to elinzanetant 120 mg or those who switched to elinzanetant 120 mg at some point during the OASIS-1 and -2 studies.

b EAIRs are defined as study size and exposure-adjusted incidence rates according to Crowe et al. (2016).^34^ EAIRs are provided per 100 person-years, where one person-year is defined as 365·25 days.

c The CI for EAIRs was calculated by stratified Wilson CI according to Yan et al. (2010).^35^

d Reported in the same participant in the placebo group.

e Data retrieved using the SMQ breast malignant tumors. The following PTs were reported: breast cancer, invasive breast carcinoma and invasive ductal breast carcinoma. One participant reported two events.

### Other safety assessments

Mean baseline values and changes from baseline at Week 12/14 and Week 52 for vital signs, and other clinical laboratory assessments are summarized in Supplementary Table S3. Overall, systemic blood pressure, serum levels of cholesterol, triglycerides, and glucose remained stable in both groups in both analyses. No clinically relevant changes were observed in serum concentrations of estradiol, progesterone, luteinizing hormone, and follicle-stimulating hormone in both groups in both analyses.

These results have been described in a graphical plain language summary in Supplementary Figure S1 and a Video.

## Discussion

In this post-hoc pooled safety analysis, greater proportion of participants (50·8% versus 43·2%) reported TEAEs with elinzanetant 120 mg during the first 12 weeks; descriptively, the EAIRs of overall TEAEs were generally comparable between the treatment groups over 52 weeks. Headache, fatigue, and somnolence were the most common treatment-related TEAEs in the elinzanetant 120 mg group, mostly reported in the first 12 weeks. Treatment-related TEAEs were also reported in the placebo group. All the studies were double-blind, and background rates of naturally occurring symptoms in the study population due to menopause symptoms or side effects of concomitant medications permitted in the study could have been coincidentally attributed to placebo by the investigator. Rates of serious TEAEs were low in both groups and generally considered not related to treatment. One serious TEAE of generalized tonic-clonic seizure occurred in one (<0·1%) participant with a history of seizures. Rare cases of seizure have been reported in patients taking elinzanetant during post-marketing surveillance. Caution is recommended in patients with a history of seizures or with conditions that potentially lower the seizure threshold.^3^ A higher proportion of women discontinued treatment due to TEAEs in the elinzanetant 120 mg group compared with placebo; discontinuation occurred mostly earlier in the treatment. Fatigue and headache were the most common TEAEs leading to treatment discontinuation in the elinzanetant group, mostly in the first 12 weeks. Most individual terms describing an adverse event leading to treatment discontinuation within each system organ class occurred at low frequencies (generally ≤1%). Overall, the TEAEs leading to treatment discontinuation did not group into any meaningful medical pattern such as specific organ class, and the absolute discontinuation rate remained low in both groups.

Liver enzyme elevations were balanced across the treatment groups. Among 1113 women who received elinzanetant 120 mg, there were zero cases detected that met the closer liver observation criteria assessed by the LSMB as being probably related to elinzanetant, with no reported concerns for serious DILI. Based on the selected population included in these clinical studies that excluded participants with history of malignancy unless in complete remission for ≥5 years, the EAIRs for malignant tumors had largely overlapping CIs between treatment groups. Additionally, no cases of endometrial hyperplasia or malignancy were reported in either group.

Most of the treatment-related TEAEs associated with elinzanetant 120 mg were mild or moderate events related to the central nervous system and were reported more frequently in the elinzanetant group than placebo. An NK-1 antagonist has shown favorable effect on sleep maintenance in primary insomnia,^36^ and clinically meaningful improvements in sleep disturbance have been reported with elinzanetant 120 mg versus placebo in SWITCH-1 and OASIS-1–4 clinical program.^7^, ^8^, ^9^^,^^13^ The impact of elinzanetant on next day driving performance and cognitive function was evaluated in a Phase I study in 64 heathy women aged 40–65 years with regular sleep patterns receiving elinzanetant 120 mg, elinzanetant 240 mg, zopiclone 7·5 mg, or placebo for 5 days in one of the four sequences. The study was powered to assess the primary endpoint of participant's ability to maintain a consistent position within the lane, measured using standard deviation of lateral position. The study found absence of clinically relevant next-morning residual effects on simulated driving performance.^37^ Additionally, in the SWITCH-1 study, no dose-dependent effect of elinzanetant was observed on number of TEAEs; while somnolence was reported more frequently in the 40-mg and 160-mg groups, it occurred at a similar incidence across all groups.^9^ While the Phase I driving simulation study provided early indication of a lack of somnolent effect the following morning, additional exploratory analyses are planned to inform the impact of bedtime dosing of elinzanetant on next day functioning. Depending on the health authority, the current advice for women who experience somnolence or fatigue during treatment with elinzanetant is to refrain from or be careful when driving or using machines until the effects have been resolved.^3^^,^^6^

Prior liver safety data from both clinical and post-marketing reports of NK-3 antagonists pavinetant and fezolinetant,^2^^,^^24^^,^^25^ provided a rationale for liver safety monitoring in the elinzanetant clinical program. The aggregated clinical data, as well as the individual case adjudications of participants with elevated liver enzymes in this exploratory pooled analysis did not detect indications of a liver safety signal, and no Hy's law cases or concerns for serious DILI were reported, consistent with what has been previously reported across the Phase I–III clinical program of elinzanetant.^33^ No hepatotoxicity warning has been issued for elinzanetant by any health authority,^3^, ^4^, ^5^, ^6^ and routine liver monitoring is not required in most countries except the US.^3^, ^4^, ^5^, ^6^ At a molecule level, elinzanetant lacks any structural features known to be associated with hepatotoxicity or any similar substructures to NK-3 receptor antagonists.^33^ Elinzanetant is also an NK-1 receptor antagonist, and approved NK-1 antagonists have not been associated with hepatotoxicity.^33^ Postmarketing surveillance will further inform the liver safety with elinzanetant in a broader population of women as the clinical studies included in this pooled safety analysis excluded women with abnormal liver parameters.

Overall, in the absence of a formal direct comparison, the overlapping CIs between the two treatment groups suggested no increased incidence of neoplasm in the elinzanetant 120 mg group in postmenopausal women included in this study as observed up to 52 weeks of treatment. However, the interpretation of these findings should be contextualized within the limitations of the enrolled population and treatment duration. Long-term safety monitoring and real-world evidence are required to fully characterize the safety profile of elinzanetant. Similarly, the comparison of overall EAIRs for malignant tumors and breast cancer with elinzanetant 120 mg and population-level incidence rates in women aged 50–64 years from the SEER program,^34^ should be interpreted with caution due to inherent differences between a selected clinical trial population with strict eligibility criteria and data from population-based registries (representative of broader population), and the exploratory nature of this analysis. Preclinical carcinogenicity studies have found no evidence of drug-related carcinogenicity with elinzanetant.^3^^,^^6^ Furthermore, there is no mechanistic risk of hormone-related cancer with elinzanetant due to modulation of neurokinin signaling without any estrogenic effect.^3^^,^^7^^,^^8^^,^^32^ To date, no endometrial malignancy has been reported in any OASIS trials, including the high-risk breast cancer population in the 52-week OASIS-4 trial,^3^^,^^13^ all of which is consistent with this new analysis of malignancies. A 3-year extension study is currently ongoing to further assess safety of elinzanetant in >90% of participants who completed 52 weeks of treatment in the OASIS-4 trial and chose to continue receiving elinzanetant.^13^

Lastly, menopause can increase the risk for the development of metabolic diseases like obesity and type 2 diabetes.^14^^,^^21^ There were no substantial changes in triglycerides, glucose, or cholesterol levels, and these remained stable over 12 and 52 weeks.

The main strength of this study was that the safety and tolerability data were obtained from a large group of participants, allowing detection of rarer safety signals that may be missed in individual trials. Secondly, the population and the study design used in these four trials were relatively similar, minimizing confounding factors such as methodological or demographic variations. Additionally, the incidence rates of TEAEs were adjusted for study sizes and exposure durations ensuring meaningful interpretation.

The main limitation of the post-hoc analyses presented was the lack of formal comparison between treatment groups due to the exploratory and non-confirmatory nature of the analysis. To help interpret safety outcomes and an assessment of overall safety and benefit–risk profile, 95% CIs for EAIRs were provided across all results for both treatment groups. Additionally, three of the four trials were placebo-controlled for only 12 weeks. Only OASIS-3 was placebo-controlled for the entire 52 weeks. Therefore, data presented here have limited statistical power to detect rare or long-latency events, such as oncological risk. Another limitation was that distinction was not made for participants experiencing a TEAE once, multiple times, or continuously, or whether it continued or worsened after switching to a different treatment in OASIS-1 and -2. Furthermore, EAIRs assume a constant hazard over time, which may potentially mask early-onset or transient risks. The lack of time-to-event analyses is a limitation to assess the clinical implications of temporal distribution of adverse events. Future studies that will include time-to-event analyses of TEAEs will allow a better understanding of the time course and outcomes of these events. Lastly, the generalizability of trial data is restricted due to trial selection criteria, for example, the age range only included women between 40 and 65 years with protocol-specified inclusion and exclusion criteria. Women with a positive pregnancy test at screening were excluded from studies. Currently, elinzanetant is contraindicated in pregnancy.^3^

In conclusion, the results from this pooled analysis support the long-term safety and tolerability of elinzanetant 120 mg in a large number of participants up to 52 weeks. The findings observed in the pooled analysis are broadly in line with those reported in the individual clinical trials, supporting the use of elinzanetant as an efficacious treatment option with a favorable safety profile for moderate-to-severe VMS associated with menopause.

## Contributors

Conceptualization: all authors. Methodology: Francuski, Lu, Djordjevic, Seitz. Data curation: Francuski, Lu, Seitz. Formal analysis: Lu, Pinkerton, Nappi, Simon, Panay. Supervision: Pinkerton, Nappi, Simon, Seitz, Panay. Writing-original draft: all authors. Writing-review & editing: all authors. Project administration: Djordjevic, Seitz. Pinkerton, Francuski, Simon, Lu, Seitz, and Panay accessed and verified the underlying data.

## Data sharing statement

Result summaries of Bayer's sponsored clinical trials in drug development phases 1, 2, 3, and 4 are provided in the Bayer Trial Finder application after marketing authorization approval in line with the position of the global pharmaceutical industry associations laid down in the “Joint Position on the Disclosure of Clinical Trial Information via Clinical Trial Registries and Databases”. In addition, results of clinical drug trials will be provided on the publicly funded website http://www.ClinicalTrials.gov and EU Clinical Trials Register in line with the applicable regulations. Bayer commits to sharing upon request from qualified scientific and medical researchers patient-level clinical trial data, study-level clinical trial data, and protocols from clinical trials in patients for medicines and indications approved in the United States (US) and European Union (EU) on or after January 01, 2014, as necessary for conducting legitimate research.

## Declaration of interests

JVP has received grants and consulting fees from Bayer Pharmaceuticals, consulting fees from Pfizer, and independent contract work for Merck & Co. REN reported personal fees from Abbott, Astellas, Bayer Healthcare, Besins Healthcare, Biocodex, Exeltis, Fidia, Ibsa, Merck & Co, Novo Nordisk, Organon, Shionogi, Theramex, and Viatris; grants from Gedeon Richter; and nonfinancial support from HRA Pharma outside the submitted work. JAS was principal investigator on the OASIS-1 trial; has received grant/research support from AbbVie, Inc., Bayer Healthcare LLC., Madorra Inc., Mylan/Viatris Inc., Myovant Sciences, Sola Pelvic Therapy, and Viking Therapeutics Inc.; has acted in a consultant/advisory capacity for Ascend Therapeutics, Bayer HealthCare Pharmaceuticals Inc., Besins Healthcare, Biote Medical, LLC, Cosette Pharmaceuticals, Inc., Duchesnay Pharmaceutical Group, Inc., Femasys Inc., IQVIA Inc., Mayne Pharma, Inc., Pfizer Inc., Penguin Bio Corp., and Vella Bioscience Inc.; has been on speaker's bureaus or received fees from Ascend Therapeutics, Bayer Healthcare LLC., Besins Healthcare, Lawley Pharmaceuticals Pty Ltd., Mayne Pharma, Inc., Myovant Sciences, Inc., and Pharmavite LLC.; NP has lectured and acted in an advisory capacity for Abbott, Astellas, Bayer, Besins, Gedeon Richter, Mithra, Novo Nordisk, SeCur, Theramex, and Viatris. CL is an employee of Bayer US LLC, USA. MF and CS are employees of Bayer AG, Berlin, Germany. SD is an employee of Bayer CC AG, Basel, Switzerland.

## Appendix A Supplementary data

Translations Abstract

Supplementary Figure and Tables

Video Safety Profile of Elinzanetant Based on Results From Multiple Clinical Studies: Quick Take Video.
