# Supplementary material for: Safety and tolerability of elinzanetant: a pooled analysis from four randomized clinical trials
Source: eClinicalMedicine. 2026 Oct 7;101:104250. doi: 10.1016/j.eclinm.2026.104250 (PMC13688456; doi:10.1016/j.eclinm.2026.104250)
Supplement: Supplementary file 1 [file mmc2.pdf]

Supplementary Information

Table of Contents

Supplementary Methods 2

Study design of SWITCH-1 and OASIS-1–3 studies 2

Exposure-adjusted incidence rate (EAIR) 2

Other safety assessments 2

Supplementary Table 1: Serious TEAEs – SWITCH-1 and OASIS-1–3 3

Supplementary Table 2: Participants with TEAEs and PTAEs in SOC: Neoplasms benign, malignant, and unspecified (including cysts and polyps) by preferred term grouping – SWITCH-1 and OASIS-1–3 4

Supplementary Table 3: Changes in blood pressure and clinical laboratory assessments from baseline to Week 12 and Week 52 – SWITCH-1 and OASIS-1–3 5

Supplementary Figure 1: Safety Profile of Elinzanetant Based on Results From Multiple Clinical Studies: A Plain Language Summary 6

References 7

Supplementary Methods

Study design of SWITCH-1 and OASIS-1–3 studies

Exclusion criteria included abnormal liver parameters; alcohol misuse; disordered proliferative endometrium; endometrial polyp, hyperplasia, or cancer; and current or history of cancer within the last 5 years (except basal and squamous cell skin tumors).[1-3] Although the Phase III OASIS-4 trial evaluated the efficacy and safety of elinzanetant, it was not included in this pooled analysis as its study population differed from those of OASIS-1, -2, and -3. Specifically, OASIS -1, -2, and -3 enrolled postmenopausal women with natural or surgical menopause, whereas OASIS-4 included women aged 18–70 years receiving endocrine therapy for hormone receptor-positive breast cancer or its prevention in those at high risk for breast cancer.[4]

SWITCH-1 was an adaptive, dose-range finding study, and eligible participants were randomized to receive elinzanetant (40, 80, 120, or 160 mg) or matching placebo once daily orally for 12 weeks.[3] For this pooled safety analysis, only data from the elinzanetant 120 mg and placebo groups are included from SWITCH-1 to correspond with the approved dose.[5] In the OASIS-1 and -2 studies, eligible participants were randomized 1:1 to receive elinzanetant 120 mg or matching placebo once daily orally for 12 weeks. After 12 weeks, all participants received elinzanetant 120 mg for additional 14 weeks.[2] In the OASIS-3 study, eligible participants were randomized 1:1 to receive elinzanetant 120 mg or matching placebo orally once daily for 52 weeks.[1] All four studies included a 4-week off-treatment follow-up.[1-3]

Exposure-adjusted incidence rate (EAIR)

EAIRs quantify how often an event (such as an adverse drug reaction) occurs relative to the total time participants are exposed to a treatment. The formula of EAIR is shown as below:

$$\textbf{Study size and exposure-adjusted IR} = \sum\left[\left(\frac{\text{number of participants with the event per study}}{\text{sum of exposure days per study}/365.25} \times 100\right) \times \left(\frac{\text{study exposure}}{\text{total exposure across studies}}\right)\right]$$

EAIR, which is study size and exposure-adjusted incidence rates, was calculated as following: for each study and treatment group, the exposure-adjusted incidence (= number of participants with event / sum of exposure days) was multiplied by the proportion of the study exposure to the total exposure across studies. Results were then added up across all studies for each treatment group, where sum of exposure days was summation of both sum of time to first TEAE for participants if an event occurred and sum of treatment duration with time at risk after treatment end for participants without TEAE; and the time at risk after treatment end was defined as time after end of treatment up to end of TEAE window (14 days) or death date, whichever was earlier. EAIRs were calculated using person-time accrued until first event or censoring. Therefore, the rates reflect incidence of first occurrence and should be interpreted as an average over time, implicitly assuming a constant rate. Results were provided per 100 person-years, where one person-year is defined as 365·25 days. The concept was derived from Crowe et al to enable comparison across trials of different lengths and design.[6] The CIs for EAIR were calculated by stratified Wilson CI according to Yan et al.[7]

Other safety assessments

Other safety assessments evaluated at baseline and at Weeks 12 and 52 included changes in blood pressure and serum levels of hormones, triglycerides, cholesterol, and glucose. All laboratory assessments were analyzed by a central laboratory. Bone mineral density was only assessed in the OASIS-3 study and is therefore not included in this pooled analysis. Bone biomarkers were evaluated across all trials; these data will be reported in a separate publication presenting the effect of elinzanetant on bone health and body composition.

Supplementary Table 1: Serious TEAEs – SWITCH-1 and OASIS-1–3

Serious TEAEs	Week 1–12		Week 1–52			
	Elinzanetant 120 mg (N=765)	Placebo (N=754)	Elinzanetant 120 mg[a] (N=1113)		Placebo (N=754)	
	n (%)		n (%)	EAIR[b] (95% CI)[c]	n (%)	EAIR[b] (95% CI)[c]
Vertigo	1 (0·1)	0	1 (<0·1)	0·22 (0·04–1·11)	0	–
Cholecystitis acute	0	1 (0·1)	0	–	1 (0·1)	0·46 (0·11–2·02)
Cholecystitis chronic	0	1 (0·1)	0	–	1 (0·1)	0·22 (0·03–1·61)
Cholelithiasis	0	1 (0·1)	2 (0·2)	0·28 (0·07–1·22)	1 (0·1)	0·46 (0·11–2·03)
Diverticulitis	1 (0·1)	0	1 (<0·1)	0·14 (0·02–0·98)	0	–
Infective exacerbation of chronic obstructive airways disease	0	1 (0·1)	0	–	1 (0·1)	0·24 (0·03–1·65)
Pneumonia	1 (0·1)	0	1 (<0·1)	0·14 (0·02–0·98)	0	–
Pyelonephritis	0	1 (0·1)	1 (<0·1)	0·14 (0·02–0·98)	1 (0·1)	0·45 (0·10–2·01)
Urosepsis	0	1 (0·1)	0	–	1 (0·1)	0·45 (0·10–2·01)
Injury	1 (0·1)	0	1 (<0·1)	0·22 (0·04–1·11)	0	–
Post procedural hemorrhage	0	1 (0·1)	0	–	1 (0·1)	0·21 (0·03–1·61)
Osteoarthritis	0	1 (0·1)	1 (<0·1)	0·22 (0·04–1·11)	1 (0·1)	0·21 (0·03–1·61)
Multiple sclerosis	1 (0·1)	0	1 (<0·1)	0·22 (0·04–1·11)	0	–
Syncope	2 (0·3)	0	2 (0·2)	0·36 (0·10–1·34)	0	–
Pulmonary embolism	2 (0·3)	0	2 (0·2)	0·28 (0·07–1·22)	0	–
Arthrodesis	1 (0·1)	0	1 (<0·1)	0·14 (0·02–0·98)	0	–
Deep vein thrombosis	1 (0·1)	0	1 (<0·1)	0·14 (0·02–0·98)	0	–
Acute myocardial infarction	–	–	1 (<0·1)	0·22 (0·04–1·11)	0	–
Abdominal pain	–	–	1 (<0·1)	0·14 (0·02–0·98)	0	–
Mechanical ileus	–	–	1 (<0·1)	0·14 (0·02–0·98)	0	–
Epiglottitis	–	–	1 (<0·1)	0·22 (0·04–1·11)	0	–
Fusobacterium infection	–	–	1 (<0·1)	0·22 (0·04–1·11)	0	–
Infected bite	–	–	0	–	1 (0·1)	0·21 (0·03–1·61)
Meningitis meningococcal	–	–	1 (<0·1)	0·22 (0·04–1·11)	0	–
Otitis externa			1 (<0·1)	0·14 (0·02–0·98)	0	–
Urinary tract infection			1 (<0·1)	0·14 (0·02–0·98)	0	–
Accident	–	–	1 (<0·1)	0·14 (0·02–0·98)	0	–
Concussion	–	–	1 (<0·1)	0·22 (0·04–1·11)	0	–
Tibia fracture	–	–	1 (<0·1)	0·14 (0·02–0·98)	0	–
Blood glucose increased	–	–	1 (<0·1)	0·22 (0·04–1·11)	0	–
Joint range of motion decreased	–	–	1 (<0·1)	0·14 (0·02–0·98)	0	–
Rotator cuff syndrome	–	–	1 (<0·1)	0·22 (0·04–1·11)	0	–
Invasive ductal breast carcinoma	–	–	1 (<0·1)	0·22 (0·04–1·11)	0	–
Encephalitis toxic	–	–	0	–	1 (0·1)	0·21 (0·03–1·61)
Generalized tonic-clonic seizure	–	–	1 (<0·1)	0·14 (0·02–0·98)	0	–
Hemiparesthesia	–	–	0	–	1 (0·1)	0·22 (0·03–1·61)
Transient ischemic attack	–	–	1 (<0·1)	0·22 (0·04–1·11)	0	–
Device loosening	–	–	1 (<0·1)	0·14 (0·02–0·98)	0	–
Bunion operation	–	–	1 (<0·1)	0·14 (0·02–0·98)	0	–
Mammoplasty	–	–	1 (<0·1)	0·14 (0·02–0·98)	0	–

N=number of subjects who received the respective treatment at any time, n=number of subjects with at least one such event.

[a]Participants exposed to elinzanetant 120 mg or those who switched to elinzanetant 120 mg at some point during the OASIS-1 and -2 studies. [b]EAIRs are defined as study size and exposure-adjusted incidence rates according to Crowe et al (2016).[6] EAIRs are provided per 100 person-years, where one person-year is defined as 365·25 days. [c]The CI for EAIRs was calculated by stratified Wilson CI according to Yan et al (2010).[35]

EAIR, exposure-adjusted incidence rate; TEAE, treatment-emergent adverse event.

Supplementary Table 2: Participants with TEAEs and PTAEs in SOC: Neoplasms benign, malignant, and unspecified (including cysts and polyps) by preferred term grouping – SWITCH-1 and OASIS-1–3

	Week 1–52			
	Elinzanetant 120 mg[a] (N=1113)		Placebo (N=754)	
Preferred Term Grouping	n (%)	EAIR[b] (95% CI)[c]	n (%)	EAIR[b] (95% CI)[c]
Participants with ≥1 event	12 (1·1)	1·89 (1·07–3·35)	4 (0·5)	1·02 (0·37–2·79)
Skin[d]	4 (0·4)	0·76 (0·31–1·83)	2 (0·3)	0·39 (0·08–1·82)
Breast[e]	3 (0·3)	0·51 (0·18–1·47)	0	–
Reproductive system[f]	3 (0·3)	0·37 (0·11–1·26)	1 (0·1)	0·19 (0·03–1·49)
Gastrointestinal[g]	1 (<0·1)	0·12 (0·02–0·86)	0	–
Other[h]	2 (0·2)	0·32 (0·08–1·18)	1 (0·1)	0·43 (0·10–1·89)

N=number of subjects who received the respective treatment at any time, n=number of subjects with at least one such event.

[a]Participants exposed to elinzanetant 120 mg or those who switched to elinzanetant 120 mg at some point during the OASIS-1 and -2 studies. [b]EAIRs are defined as study size and exposure-adjusted incidence rates according to Crowe et al (2016).[6] EAIRs are provided per 100 person-years, where one person-year is defined as 365·25 days. [c]The CI for EAIRs was calculated by stratified Wilson CI according to Yan et al (2010).[7] [d]Includes preferred terms basal cell carcinoma, Bowen's disease, melanocytic naevus, squamous cell carcinoma of skin. [e]Includes preferred terms breast cancer, fibroadenoma of breast, intraductal papilloma of breast, invasive breast carcinoma, invasive ductal breast carcinoma. [f]Includes preferred terms leiomyoma, uterine leiomyoma. [g]Includes preferred terms adenocarcinoma of colon. [h]Includes preferred terms benign neoplasm, hypergammaglobulinemia benign monoclonal, lipoma.
CI, confidence interval; EAIR, exposure-adjusted incidence rate; PTAE, post-treatment adverse event; SOC, system organ class; TEAE, treatment-emergent adverse event.

Supplementary Table 3: Changes in blood pressure and clinical laboratory assessments from baseline to Week 12 and Week 52 – SWITCH-1 and OASIS-1–3

	Week 1–12[a]		Week 1–52[b,c]
	Elinzanetant 120 mg Mean (SD), n	Placebo Mean (SD), n	Elinzanetant 120 mg Mean (SD), n
**Systolic blood pressure (mmHg)**			
Baseline	119·8 (10·3), 765	120·1 (10·7), 754	119·9 (10·4), 1113
Change from baseline at Week 12/14	−0·9 (10·0), 671	−1·3 (10·8), 671	−1·0 (10·1), 998
Change from baseline at Week 52	–	–	0·7 (9·9), 243
**Diastolic blood pressure (mmHg)**			
Baseline	76·2 (7·2), 765	76·4 (7·3), 754	76·2 (7·1), 1113
Change from baseline at Week 12/14	−1·2 (7·5), 671	−0·2 (7·2), 671	−1·0 (7·3), 998
Change from baseline at Week 52	–	–	−0·3 (7·7), 243
**Glucose (mg/dL)**			
Baseline	93·95 (16·56), 760	95·64 (22·76), 754	94·21 (16·94), 1108
Change from baseline at Week 12/14	2·60 (20·49), 650	0·86 (19·67), 659	2·66 (20·69), 972
Change from baseline at Week 52	–	–	0·57 (19·78), 239
**Cholesterol (mg/dL)**			
Baseline	213·14 (40·08), 713	216·70 (38·83), 707	214·60 (39·53), 1061
Change from baseline at Week 12/14	1·02 (26·10), 613	−3·07 (26·81), 626	0·43 (27·46), 939
Change from baseline at Week 52	–	–	4·03 (32·92), 239
**Triglycerides (mg/dL)**			
Baseline	135·77 (78·86), 713	142·04 (84·85), 707	138·22 (78·45), 1061
Change from baseline at Week 12/14	−7·50 (57·34), 613	5·61 (85·18), 625	−4·13 (77·50), 939
Change from baseline at Week 52	–	–	−1·77 (68·93), 239
**Estradiol (pg/mL)**			
Baseline	14·01 (33·10), 713	14·08 (40·45), 706	13·48 (30·18), 1061
Change from baseline at Week 12/14	−1·15 (36·57), 613	2·02 (44·09), 625	−1·37 (34·77), 939
Change from baseline at Week 52	–	–	−5·04 (38·57), 240
**FSH (U/L)**			
Baseline	77·4 (30·1), 737	77·7 (30·2), 727	77·5 (30·3); 1085
Change from baseline at Week 12/14	1·1 (16·6), 613	−1·3 (15·8), 621	1·0 (16·4), 938
Change from baseline at Week 52	–	–	2·9 (20·2), 239
**LH (U/L)**			
Baseline	41·9 (15·1), 713	41·8 (14·7), 707	42·1 (15·0), 1061
Change from baseline at Week 12/14	−1·1 (10·8), 613	−0·9 (9·0), 624	−1·3 (10·5), 938
Change from baseline at Week 52	–	–	−1·7 (9·8), 240
**Progesterone (µg/L)**			
Baseline	0·13 (0·86), 713	0·13 (0·58), 706	0·12 (0·71), 1061
Change from baseline at Week 12/14	−0·02 (0·93), 613	0·01 (0·74), 625	−0·01 (0·76), 937
Change from baseline at Week 52	–	–	−0·02 (0·56), 240

[a]For elinzanetant 120 mg and placebo groups (Week 1–12), integrated analysis visit 12/14 refers only to study visit week 12; treatment measurements are only presented to this time point. [b]Participants exposed to elinzanetant 120 mg or those who switched to elinzanetant 120 mg at some point during the OASIS-1 and -2 studies. [c]In order to mirror the time relative to the start of elinzanetant treatment, treatment switchers visit Week 26 is presented by integrated analysis visit week 12/14 for elinzanetant 120 mg (Week 1–52) group.
FSH, follicle stimulating hormone; LH, luteinizing hormone.

Supplementary Figure 1: Safety Profile of Elinzanetant Based on Results From Multiple Clinical Studies: A Plain Language Summary

Safety Profile of Elinzanetant Based on Results From Multiple Clinical Studies: A Plain Language Summary

JoAnn V. Pinkerton, MD[1]; Maja Francuski, MD[2]; Rossella E. Nappi, MD[3,4]; James A. Simon, MD[5];
Chenshuang Lu, MS[6]; Senka Djordjevic, MD[7]; Christian Seitz, MD[2,8]; Nick Panay, MBBS[9]

[1]Department of Obstetrics and Gynecology, Division Midlife Health, University of Virginia Health, Charlottesville, Virginia, USA; [2]Bayer AG, Berlin, Germany; [3]Department of Clinical, Surgical, Diagnostic and Pediatric Sciences, University of Pavia, Pavia, Italy; [4]Research Center for Reproductive Medicine, Gynecological Endocrinology and Menopause, IRCCS San Matteo Foundation, Pavia, Italy; [5]George Washington University, IntimMedicine Specialists, Washington, DC, USA; [6]Bayer US LLC, Whippany, New Jersey, USA; [7]Bayer CC AG, Basel, Switzerland; [8]Charité - Universitätsmedizin Berlin, Berlin, Germany; [9]Queen Charlotte's and Chelsea Hospital, Imperial College London, London, England

This is a plain language summary of the safety profile of elinzanetant based on results from multiple clinical studies evaluating its use in women with natural or surgical menopause experiencing moderate-to-severe hot flashes.

WHY WAS THE STUDY CARRIED OUT?

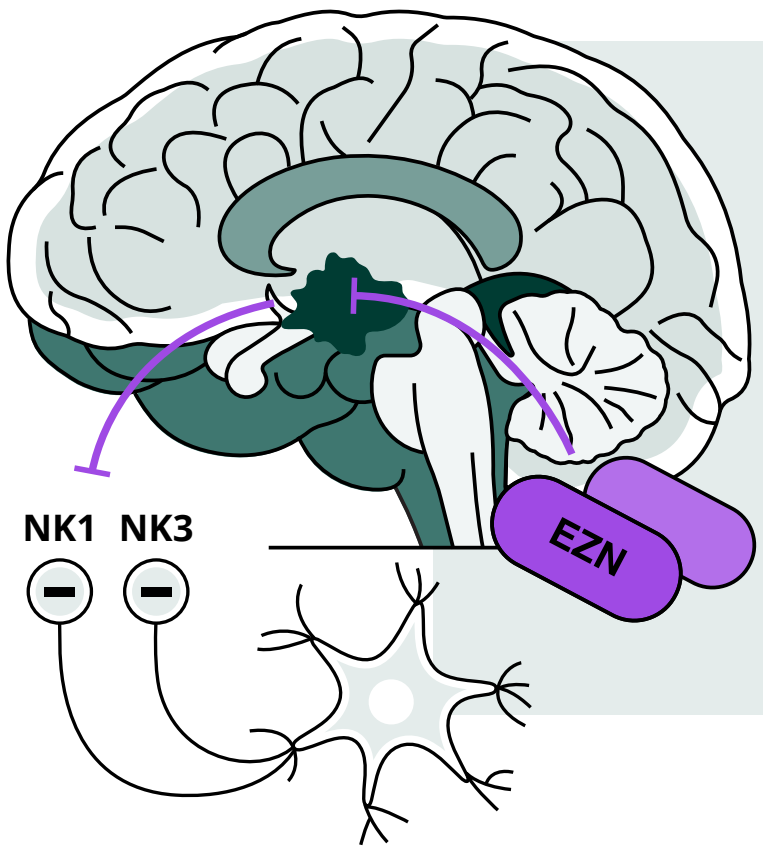

Elinzanetant is a **hormone-free** treatment targeting the source of **hot flashes** in the brain.

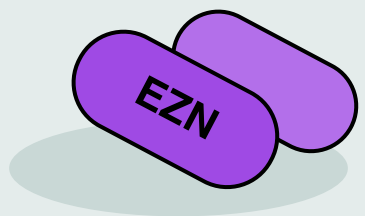

Elinzanetant blocks two brain signal pathways, **NK-1 and NK-3**, linked to hot flashes and sleep disturbances during menopause.

Elinzanetant is currently approved for the treatment of hot flashes associated with menopause, and also for the treatment of hot flashes caused by endocrine therapy for breast cancer in multiple countries.[1–4]

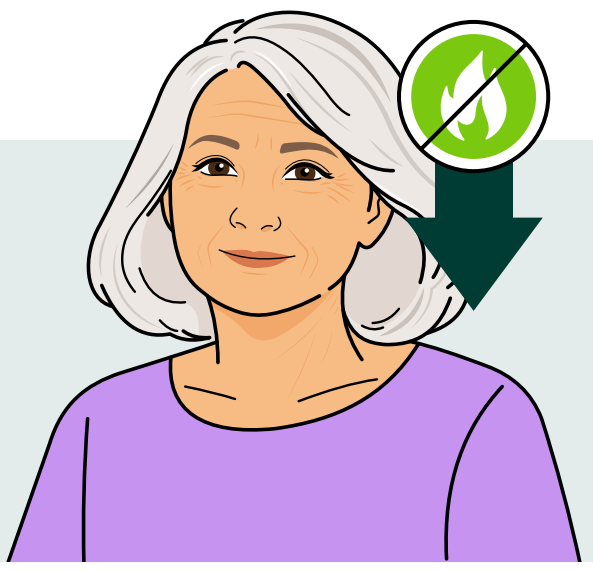

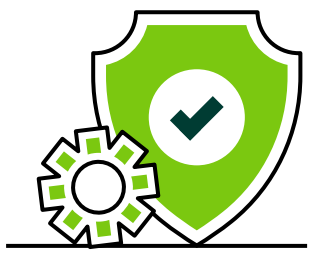

The side effects seen with elinzanetant in individual clinical studies were mainly mild to moderate, the most common being headache, fatigue, and sleepiness.

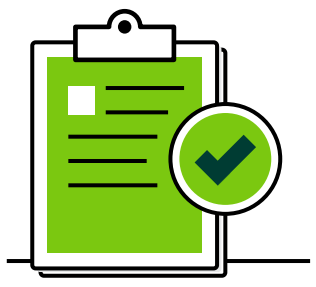

Safety data were pooled from multiple clinical studies to allow detection of rare side effects in more than 1500 women.

HOW WAS THE STUDY CARRIED OUT?

Data were pooled from 4 clinical studies:
SWITCH-1, OASIS-1, OASIS-2, and OASIS-3.

Who was included in the study?

- 1519 women with natural or surgical menopause
- Experiencing moderate-to-severe hot flashes
- 40–65 years of age

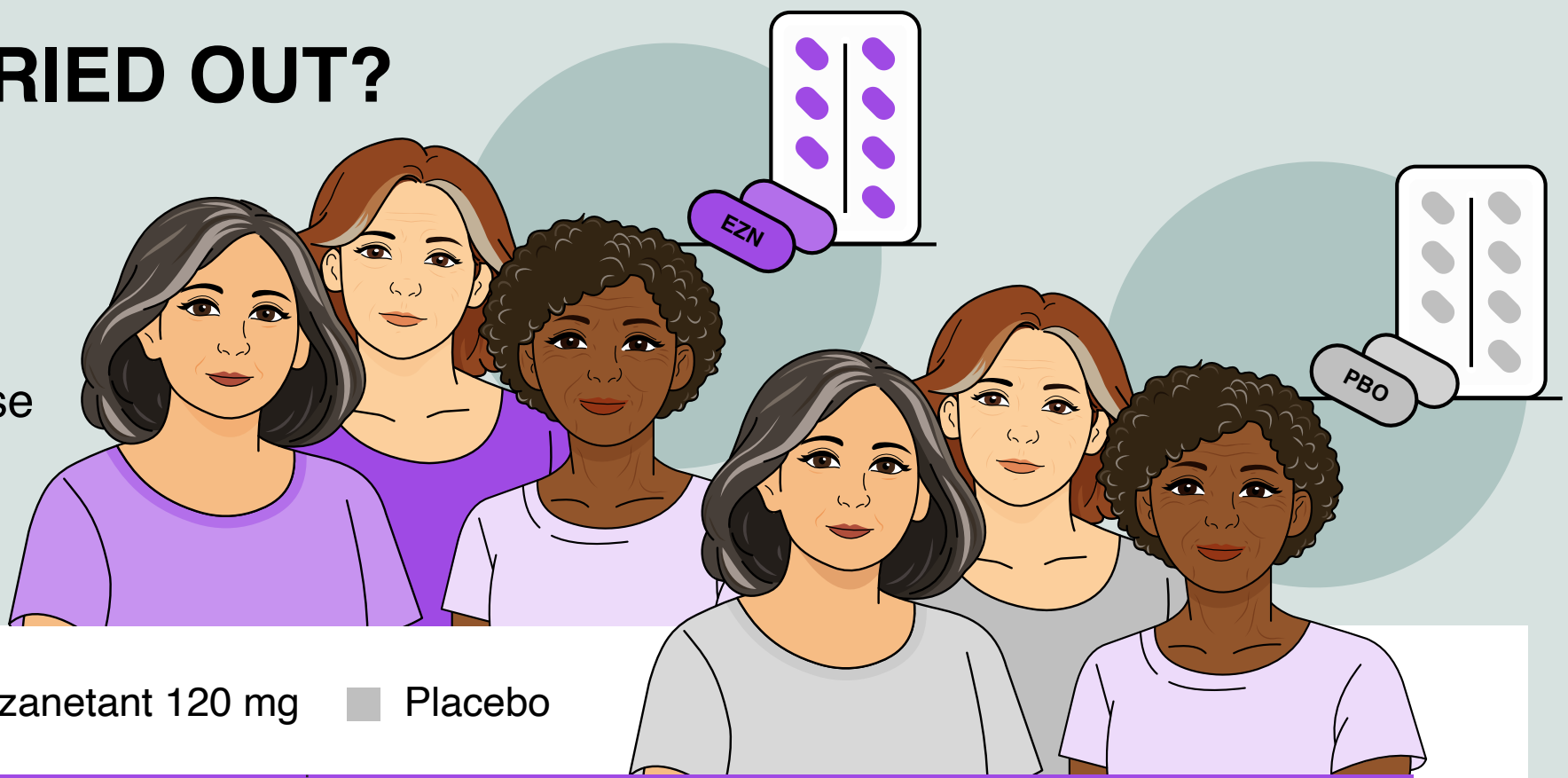

How were the studies set up?

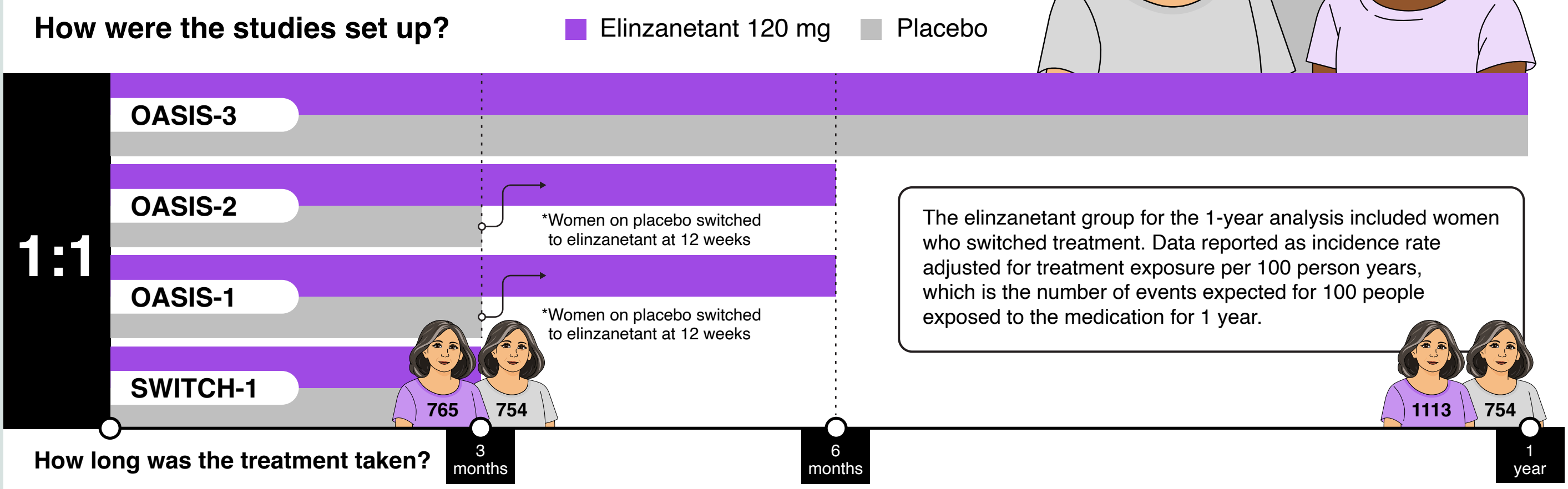

Side effects and other areas of interest were assessed continuously. Results are presented from analyses over 3 months and 1 year.
These analyses were conducted to explore patterns in the data and did not formally compare differences between elinzanetant and placebo groups.

WHAT WERE THE OVERALL RESULTS OF THE STUDY?

POOLED TRIALS
- SWITCH-1
- OASIS-1
- OASIS-2
- OASIS-3

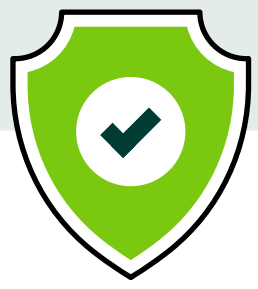

NO NEW SIDE EFFECTS WERE DETECTED BEYOND THOSE ALREADY REPORTED

Side effects were monitored in

1113 women

taking elinzanetant up to 1 year; majority (>90%) were mild to moderate.

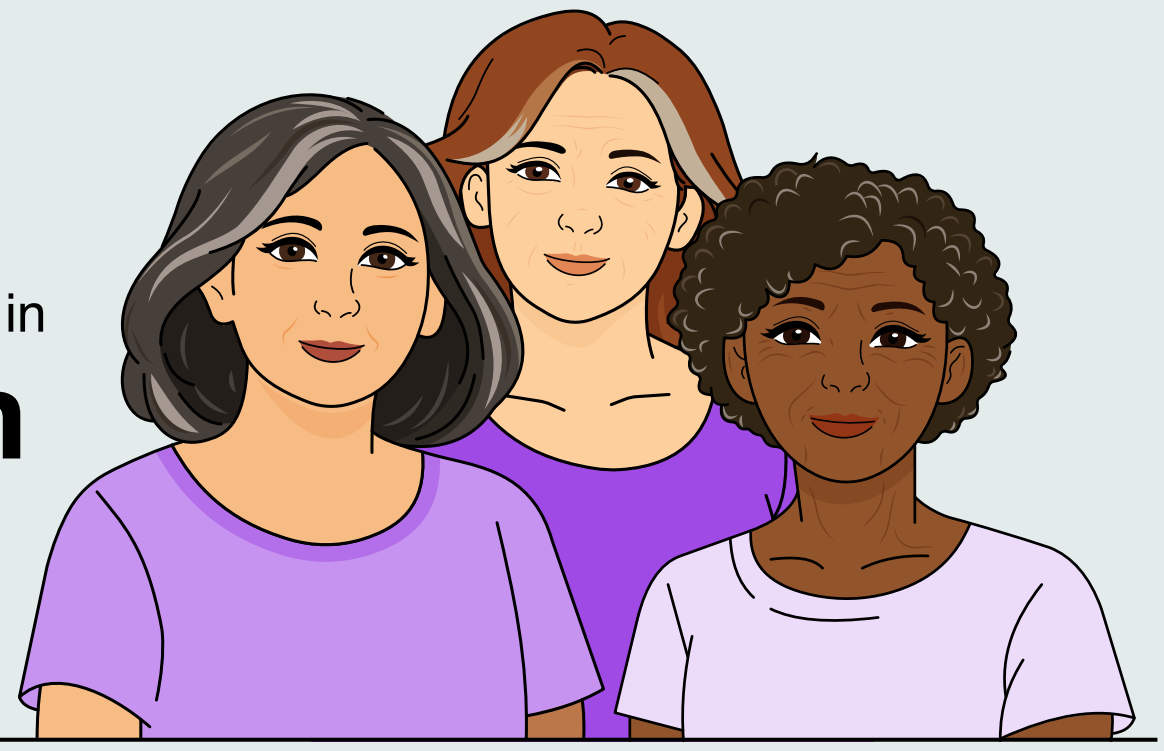

Other areas of interest

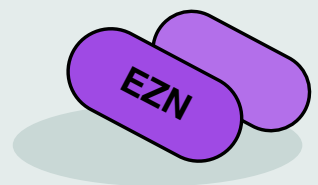

- Women taking elinzanetant in the studies did not experience serious liver problems.
- Within the selected population of women in the studies, no increased occurence of cancer was observed with elinzanetant up to 1 year based on incidence rates.

REAL WORLD EVIDENCE WILL FURTHER INFORM THE SAFETY PROFILE OF ELINZANETANT IN A BROADER POPULATION OF WOMEN.

SIDE EFFECTS

More women in the elinzanetant group reported a side effect than placebo (51% versus 43%) during the first 3 months.

The overall rate of side effects was generally comparable in both groups over 1 year.

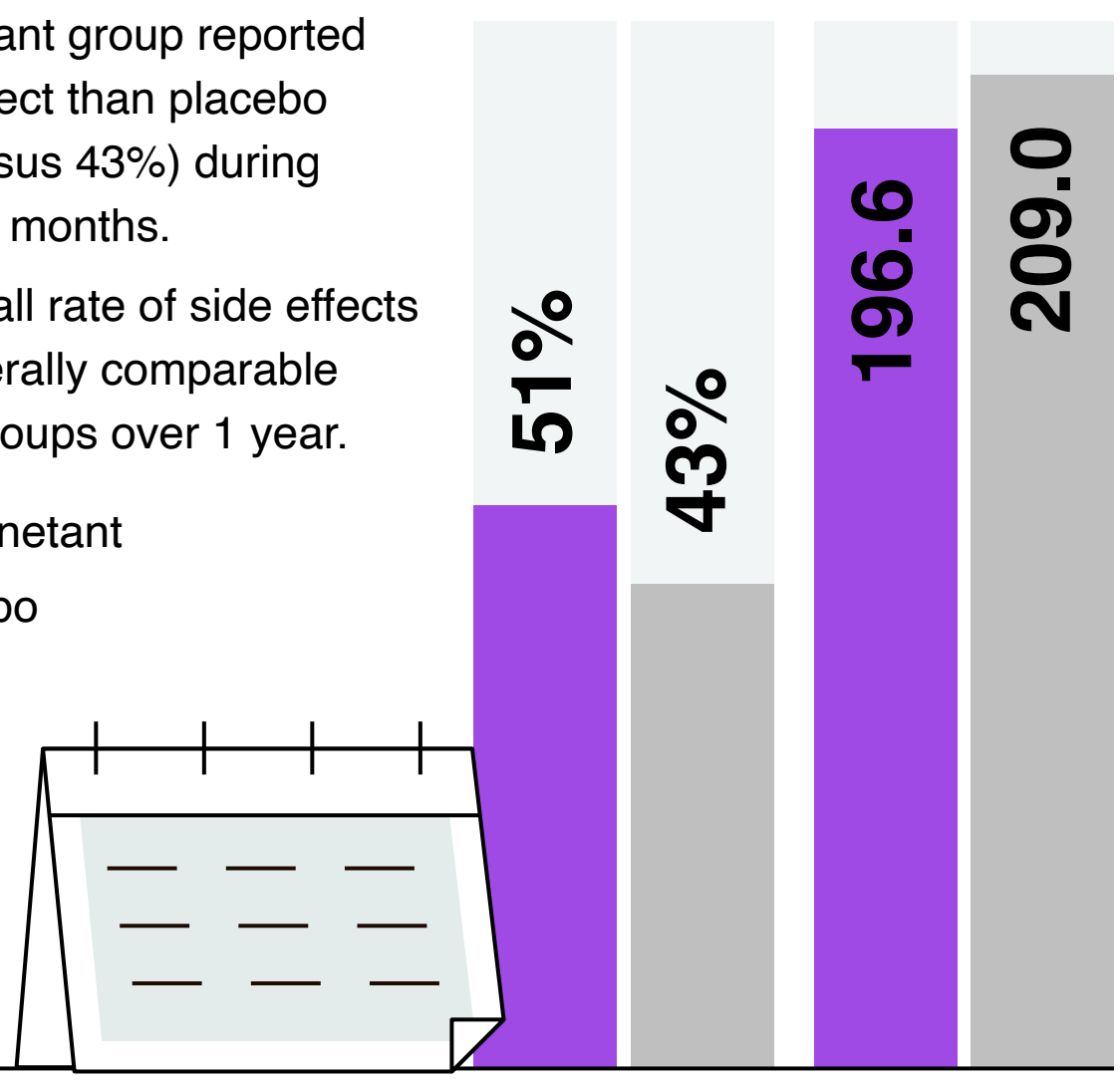

Most common side effects with elinzanetant:

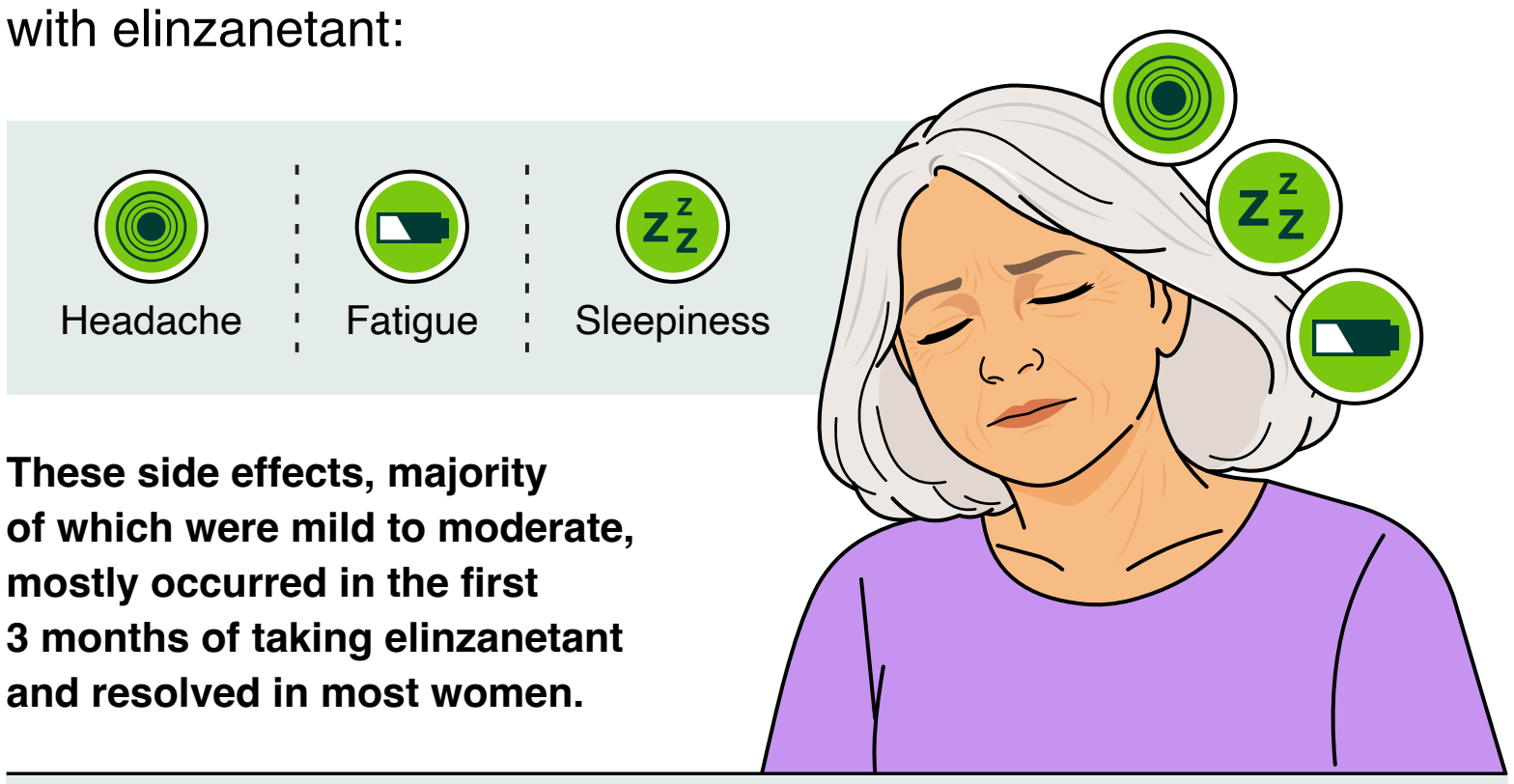

These side effects, majority of which were mild to moderate, mostly occurred in the first 3 months of taking elinzanetant and resolved in most women.

A small and dedicated study reported that taking elinzanetant at bedtime did not have an impact on driving the next morning.[4]
Women are advised to be careful or refrain from driving or using machines if they experience fatigue or sleepiness during treatment with elinzanetant.[1,2]

*Women across the pooled studies were treated for different lengths of time. How often a side effect occurred was calculated based on the total time women were exposed to treatment. The 1-year results were reported as the number of side effects expected for every 100 people treated for 1 year (rate).

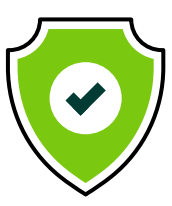

Few (7.5%) women stopped taking elinzanetant due to side effects over 1 year; this percentage was higher than with placebo (4.1%).

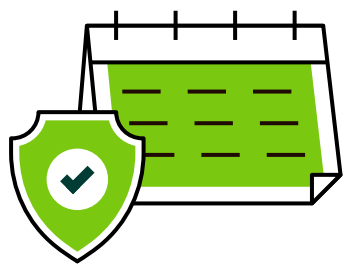

Fatigue (1.7%) and headache (1.4%) were the most common reasons for stopping treatment with elinzanetant in the first 3 months. After 3 months, there was no discontinuation due to fatigue and only 1 discontinuation due to headache.

Rare cases of seizure have been reported in patients taking elinzanetant. Caution is recommended in patients with a history of seizures or with conditions that can make you more susceptible to seizure.[1,3,4]

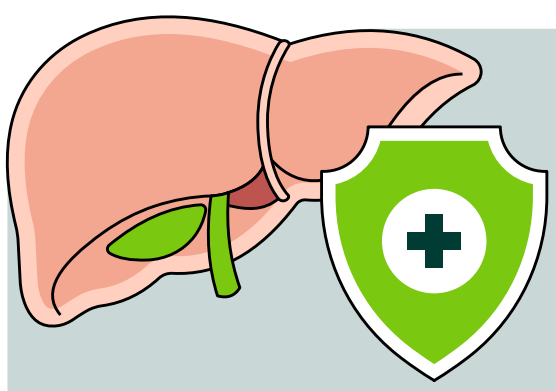

OVERALL, NO SERIOUS LIVER PROBLEMS WERE FOUND IN THIS STUDY.
(over 3 months and 1 year)

LIVER SAFETY OF ELINZANETANT

Based on the absence of serious liver problems seen in clinical trials, an independent group of liver safety experts concluded that routine blood tests to check liver health are not needed for elinzanetant.

Blood tests to check your liver are not required in most countries,[2–4] *except for the US, where the FDA requires liver monitoring before and after 3 months of starting treatment with elinzanetant.*[1]

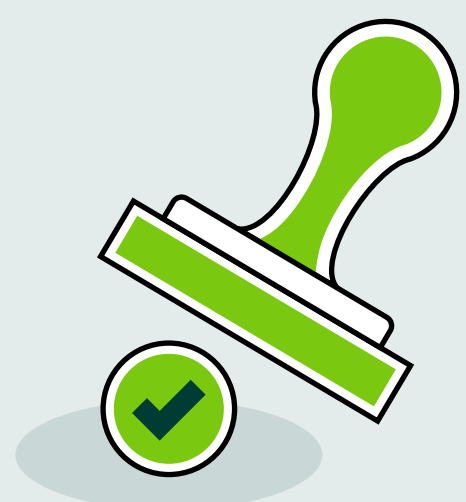

NO INCREASED OCCURRENCE OF MALIGNANCY IN WOMEN INCLUDED IN THE STUDIES

While findings from clinical studies with strict eligibility criteria may not be fully generalizable to the broader population of women represented in real-world data, overall, the rate of developing any type of malignant cancer after taking elinzanetant was in line with the background rate of developing cancer in US women aged 50 to 64 years.

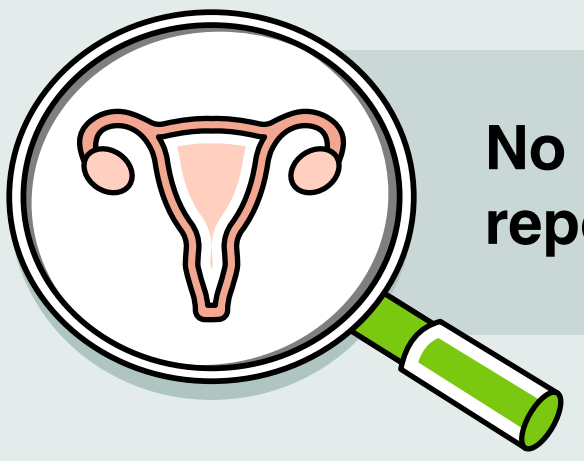

No cases of endometrial cancer reported in any treatment group.

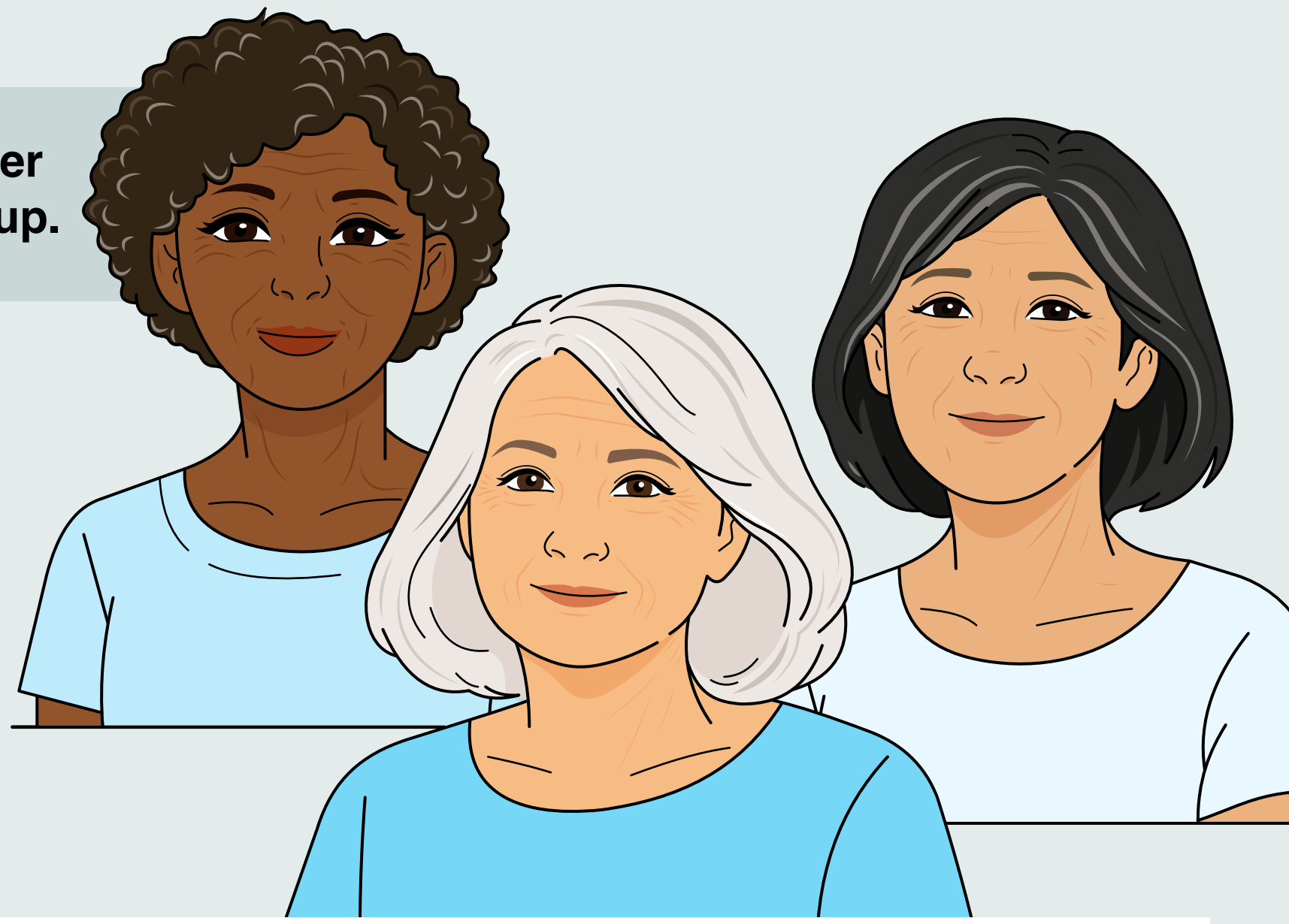

2 breast cancer cases in the elinzanetant group (in line with background rate in US women aged 50–64 years).

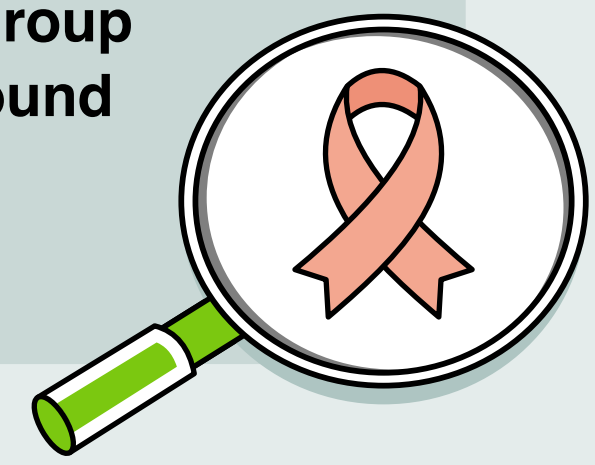

LAB TESTS

No clinically relevant change in:

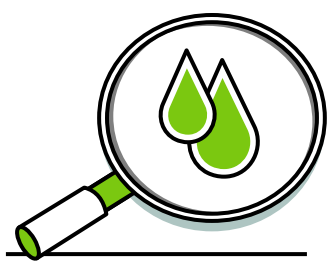

Cholesterol
Triglycerides

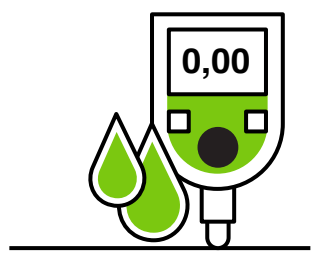

Glucose

WHAT DO THESE RESULTS MEAN?

These results support the use of elinzanetant in women with moderate-to-severe hot flashes.

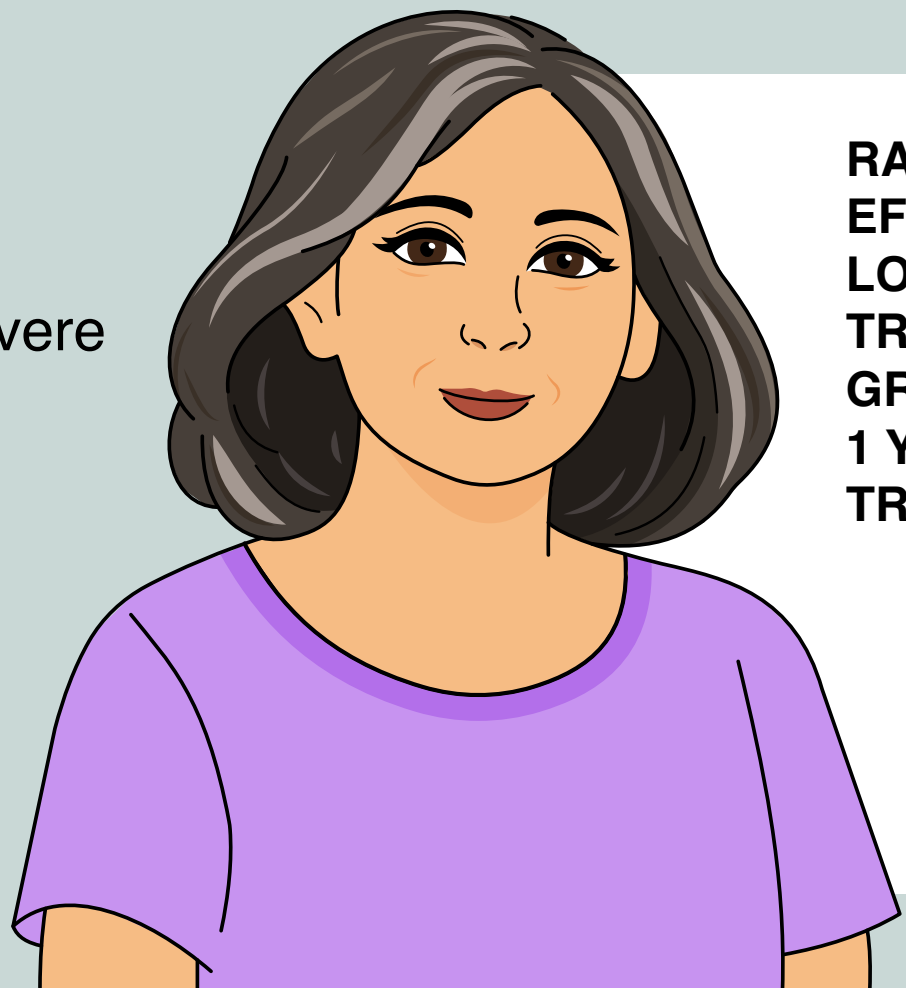

DATA FROM A LARGE AND DIVERSE GROUP OF WOMEN

RATE OF SIDE EFFECTS WAS LOW IN BOTH TREATMENT GROUPS OVER 1 YEAR OF TREATMENT

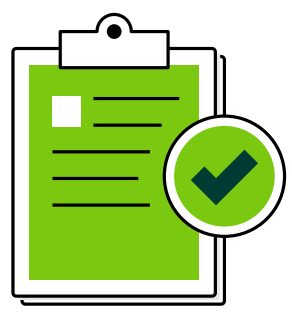

NO SERIOUS LIVER PROBLEMS IN WOMEN INCLUDED IN THE STUDIES

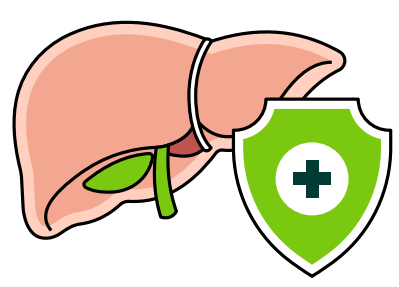

NO INCREASED OCCURRENCE OF ENDOMETRIAL AND BREAST CANCER IN STUDY PARTICIPANTS

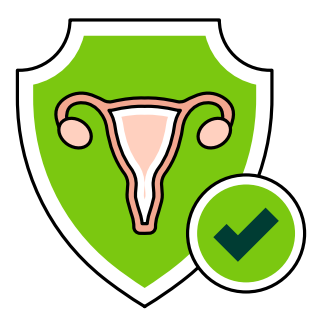

1. LYNKUET® (elinzanetant) [Prescribing Information]. Whippany, NJ: Bayer HealthCare Pharmaceuticals, Inc.; August 2026. **2.** Lynkuet 60 mg soft capsules. EMA SmPC. https://www.ema.europa.eu/en/medicines/human/EPAR/lynkuet. **3.** Lynkuet 60 mg soft capsules. UK SmPC. **4.** LYNKUET® (elinzanetant) Product Monograph. August 2026. https://www.bayer.com/sites/default/files/lynkuet-pm-en.pdf. **5.** Francke K, Klein S, Burke J, Schultze-Mosgau MH, Kay G. The Effects of Elinzanetant on Simulated Driving Performance in Healthy Women: A Randomized Phase I Study. *Clin Transl Sci* 2025; 18(7): e70282.

Disclaimer:

This plain language summary represents the opinions of the authors. For a full list of declarations, including funding and author disclosure statements, and copyright information, please see the full text online.

For more information on elinzanetant, please access the Prescribing Information or Summary of Product Characteristics.

References

1. Panay N, Joffe H, Maki PM, et al. Elinzanetant for the Treatment of Vasomotor Symptoms Associated With Menopause: A Phase 3 Randomized Clinical Trial. *JAMA Intern Med* 2025.
2. Pinkerton JV, Simon JA, Joffe H, et al. Elinzanetant for the Treatment of Vasomotor Symptoms Associated With Menopause: OASIS 1 and 2 Randomized Clinical Trials. *JAMA* 2024; **332**(16): 1343-54.
3. Simon JA, Anderson RA, Ballantyne E, et al. Efficacy and safety of elinzanetant, a selective neurokinin-1,3 receptor antagonist for vasomotor symptoms: a dose-finding clinical trial (SWITCH-1). *Menopause* 2023; **30**(3): 239-46.
4. Cardoso F, Parke S, Brennan DJ, et al. Elinzanetant for Vasomotor Symptoms from Endocrine Therapy for Breast Cancer. *N Engl J Med* 2025; **393**(8): 753-63.
5. LYNKUET® (elinzanetant) [Prescribing Information]. Whippany, NJ: Bayer HealthCare Pharmaceuticals, Inc.; August 2026.
6. Crowe B, Chuang-Stein C, Lettis S, Brueckner A. Reporting Adverse Drug Reactions in Product Labels. *Ther Innov Regul Sci* 2016; **50**(4): 455-63.
7. Yan X, Su XG. Stratified Wilson and Newcombe Confidence Intervals for Multiple Binomial Proportions. *Statistics in Biopharmaceutical Research* 2010; **2**(3): 329-35.
