# Supplementary material for: Safety and tolerability of elinzanetant: a pooled analysis from four randomized clinical trials
Source: eClinicalMedicine. 2026 Oct 7;101:104250. doi: 10.1016/j.eclinm.2026.104250 (PMC13688456; doi:10.1016/j.eclinm.2026.104250)
Supplement: Supplementary file 2 [file mmc1.docx]

**Résumé**

**Contexte** L’élinzanétant est un double antagoniste des récepteurs de la neurokinine (NK)-1 et NK-3. Il a été approuvé pour le traitement des symptômes vasomoteurs (SMV) modérés à sévères associés à la ménopause, ou de ceux causés par une hormonothérapie adjuvante pour le traitement du cancer du sein.

**Méthodes** Cette analyse regroupée des données de sécurité a inclus les données issues de quatre études randomisées, contrôlées par placebo : l’étude de phase IIb SWITCH-1 (2018-2019) et les études de phase III OASIS-1-3 (2021-2024). Ces quatre études internationales, multicentriques et en double aveugle incluaient des femmes ménopausées âgées de 40 à 65 ans présentant des SMV modérés à sévères liés à une ménopause naturelle ou chirurgicale. Les données ont été analysées de manière exploratoire. Les évaluations comprenaient la fréquence des événements indésirables apparus sous traitement (EIAT) sur 12 et 52 semaines, ainsi que les taux d’incidence ajustés en fonction de l’exposition (TIAE), avec leurs intervalles de confiance [IC] à 95 % sur 52 semaines. Les TIAE ont été calculés en pondérant le nombre d’événements en fonction du temps d’exposition total (personne-années) dans l’ensemble des études. La sécurité hépatique et l’incidence des néoplasies ont également été évaluées.

**Résultats** L’ensemble d’analyse regroupée des données de sécurité sur 12 semaines comprenait 1 519 participantes : 765 dans le groupe élinzanétant 120 mg et 754 dans le groupe placebo. L’ensemble d’analyse regroupée des données de sécurité sur 52 semaines comprenait 1 113 participantes dans le groupe élinzanétant 120 mg et 754 dans le groupe placebo. Des EIAT sont survenus chez 50,8 % et 43,2 % des participantes dans les groupes élinzanétant 120 mg et placebo, respectivement, sur 12 semaines ; les TIAE étaient de 196,61 (IC à 95 % : 181,33-213,18) et 209,00 (IC à 95 % : 188,96-231,15) dans les groupes élinzanétant et placebo, respectivement, sur 52 semaines. Les EIAT les plus fréquemment rapportés avec l’élinzanétant étaient les céphalées, la fatigue et la somnolence. Le taux d’arrêt du traitement était faible avec l’élinzanétant (7,8 %), mais plus élevé qu’avec le placebo (3,6 %) sur 12 semaines ; les TIAE étaient de 14,95 (IC à 95 % : 11,99-18,62) et de 10,96 (IC à 95 % : 7,87-15,27), respectivement, sur 52 semaines. Aucun cas de loi de Hy ni aucune indication d’atteinte cholestatique n’ont été observés. Les TIAE de tumeurs malignes présentaient des IC se chevauchant entre les groupes de traitement. Aucune hyperplasie ni tumeur maligne de l’endomètre n’a été rapportée dans l’un ou l’autre groupe.

**Interprétation** Dans cette analyse exploratoire regroupée des données de sécurité, le profil global de sécurité de l’élinzanétant a semblé favorable chez 1 113 femmes. Des analyses futures du délai jusqu’à la survenue des EIAT ainsi que des données post-commercialisation issues de la pratique clinique courante sont nécessaires afin de mieux caractériser le profil de sécurité à long terme de l’élinzanétant.

**Financement** Bayer.

**Zusammenfassung**

**Hintergrund** Elinzanetant ist ein dualer Neurokinin(NK)-1- und NK-3-Rezeptorantagonist, der zur Behandlung von mittelschweren bis schweren vasomotorischen Symptomen (VMS) im Zusammenhang mit den Wechseljahren oder solchen, die durch eine adjuvante endokrine Therapie bei Brustkrebs verursacht werden, zugelassen ist.

**Methoden** In dieser gepoolten Sicherheitsanalyse wurden Sicherheitsdaten aus vier randomisierten, placebokontrollierten Studien zusammengeführt: der Phase-IIb-Studie SWITCH-1 (2018–2019) und den Phase-III-Studien OASIS-1–3 (2021–2024). Alle vier Studien waren multizentrische, multinationale Doppelblindstudien, an denen postmenopausale Frauen im Alter von 40 bis 65 Jahren teilnahmen, die aufgrund einer natürlichen oder chirurgischen Menopause unter mittelschweren bis schweren vasomotorischen Symptomen litten. Die Daten wurden explorativ analysiert. Zu den Beurteilungen gehörten die Häufigkeit von unter der Behandlung aufgetretenen unerwünschten Ereignissen (TEAEs) über 12 und 52 Wochen sowie die expositionsbereinigten Inzidenzraten (EAIRs, mit 95%-Konfidenzintervallen [KI]) über 52 Wochen. Die EAIRs wurden berechnet, indem die Ereignisanzahlen gegen die gesamte Expositionszeit (Personenjahre) über alle Studien hinweg gewichtet wurden. Zusätzlich wurden die Lebersicherheit und die Inzidenz von Neoplasmen bewertet.

**Ergebnisse** Die gepoolte Sicherheitsanalyse über 12 Wochen umfasste 1519 Teilnehmerinnen: 765 Teilnehmerinnen in der Elinzanetant-120 mg-Gruppe und 754 Teilnehmerinnen in der Placebo-Gruppe. Die gepoolte Sicherheitsanalyse über 52 Wochen umfasste 1113 Teilnehmerinnen in der Elinzanetant-120 mg-Gruppe und 754 Teilnehmerinnen in der Placebo-Gruppe. TEAEs traten bei 50,8 % der Teilnehmerinnen in der Elinzanetant-120 mg-Gruppe und bei 43,2 % der Teilnehmerinnen in der Placebo-Gruppe über einen Zeitraum von 12 Wochen auf; die EAIRs betrugen 196,61 (95 % KI 181,33–213,18) in der Elinzanetant-Gruppe und 209,00 (95 % KI: 188,96–231,15) in der Placebo-Gruppe über einen Zeitraum von 52 Wochen. Die am häufigsten berichteten unerwünschten Ereignisse im Zusammenhang mit Elinzanetant waren Kopfschmerzen, Müdigkeit und Schläfrigkeit. Die Abbruchrate der Behandlung war unter Elinzanetant niedrig (7,8 %), aber höher als unter Placebo (3,6 %) über 12 Wochen; die EAIRs betrugen 14,95 (95 % KI 11,99–18,62) bzw. 10,96 (95 % KI 7,87–15,27) über 52 Wochen. Es gab keine Hy’s-Law-Fälle und auch keine Hinweise auf cholestatische Schädigungen. Die EAIRs maligner Tumoren wiesen überlappende KI zwischen den Behandlungsgruppen auf. In keiner der beiden Gruppen wurde über Endometriumhyperplasie/Malignität berichtet.

**Interpretation** Das allgemeine Sicherheitsprofil von Elinzanetant erschien in dieser explorativen gepoolten Sicherheitsanalyse bei 1113 Frauen günstig. Zukünftige Zeit-bis-Ereignis-Analysen von TEAEs und Daten aus der Praxis nach der Markteinführung sind erforderlich, um die langfristige Sicherheit und Verträglichkeit von Elinzanetant besser zu charakterisieren.

**Finanzierung** durch Bayer.

**Resumen**

**Descripción general** Elinzanetant es un antagonista dual de los receptores de neurocinina (NK)-1 y NK-3 aprobado para el tratamiento de los síntomas vasomotores (SVM) de moderados a graves asociados a la menopausia o causados por la terapia endocrina adyuvante para el tratamiento del cáncer de mama.

**Métodos** En este análisis de seguridad combinado, se agruparon los datos de seguridad de cuatro estudios aleatorizados, controlados con placebo: el ensayo SWITCH-1 de fase IIb (2018-2019) y los ensayos OASIS-1–3 de fase III (2021-2024). Los cuatro estudios fueron ensayos multicéntricos, multinacionales, a doble ciego en los que participaron mujeres posmenopáusicas de entre 40 y 65 años de edad que presentaban SVM de moderados a graves debido a la menopausia natural o quirúrgica. Los datos se analizaron de forma exploratoria. Las evaluaciones incluyeron la frecuencia de eventos adversos emergentes del tratamiento (EAET) durante 12 y 52 semanas y las tasas de incidencia ajustadas por exposición (TIAE, con intervalos de confianza [IC] del 95 %) durante 52 semanas. Las TIAE se calcularon ponderando los recuentos de eventos frente al tiempo total de exposición (años-persona) en los estudios. Además, se evaluaron la seguridad hepática y la incidencia de neoplasias.

**Resultados** El conjunto de análisis de seguridad combinado de 12 semanas incluyó 1519 participantes: 765 participantes en el grupo de 120 mg de elinzanetant y 754 participantes en el grupo de placebo. El conjunto de análisis de seguridad combinado de 52 semanas incluyó 1113 participantes en el grupo de 120 mg de elinzanetant y 754 participantes en el grupo de placebo. EAET ocurrieron en el 50.8 % y el 43.2 % de los participantes de los grupos de 120 mg de elinzanetant y placebo, respectivamente, durante 12 semanas; las TIAE fueron 196.61 (IC del 95 %: 181.33–213.18) y 209·00 (IC del 95 %: 188.96–231.15) en los grupos de elinzanetant y placebo, respectivamente, durante 52 semanas. Los EAET más frecuentes notificados con elinzanetant fueron dolor de cabeza, fatiga y somnolencia. La interrupción del tratamiento fue baja con elinzanetant (7.8 %), pero superior al placebo (3.6 %) durante 12 semanas; las TIAE fueron de 14.95 (IC del 95 %: 11.99–18.62) y 10.96 (IC del 95 %: 7.87–15.27), respectivamente, durante 52 semanas. No hubo casos de la Ley de Hy ni indicios de lesión colestásica. Las TIAE de los tumores malignos presentaron IC superpuestos entre los grupos de tratamiento. No se notificó hiperplasia/neoplasia maligna de endometrio en ninguno de los grupos.

**Interpretación** El perfil de seguridad general de elinzanetant pareció favorable en 1113 mujeres en función de este análisis exploratorio de seguridad combinado. Está justificada la realización de futuros análisis del tiempo hasta el evento de los EAET y de los datos posteriores a la comercialización del mundo real para caracterizar mejor el perfil de seguridad a largo plazo de elinzanetant.

**Financiación** Bayer.
